# Genome-wide Evidence of Host Specialization in Wild and Farmland Populations of the Fungal Leaf Spot Pathogen, *Cercospora beticola*

**DOI:** 10.1093/gbe/evaf053

**Published:** 2025-04-28

**Authors:** Demetris Taliadoros, Lizel Potgieter, Amar Dhiman, Nathan A Wyatt, Mark McMullan, Christian Jung, Melvin D Bolton, Eva H Stukenbrock

**Affiliations:** Environmental Genomics, Max Planck Institute for Evolutionary Biology, Plön 24306, Germany; Environmental Genomics group, Botanical Institute, Christian-Albrechts University of Kiel, Kiel 24118, Germany; Environmental Genomics, Max Planck Institute for Evolutionary Biology, Plön 24306, Germany; Environmental Genomics group, Botanical Institute, Christian-Albrechts University of Kiel, Kiel 24118, Germany; Plant Breeding Institute, Christian-Albrechts University of Kiel, Kiel 24118, Germany; USDA-ARS Edward T. Schafer Agricultural Research Center, Sugarbeet Research Unit, Fargo, ND, USA; Earlham Institute, Norwich Research Park, Norwich NR4 7UZ, UK; Plant Breeding Institute, Christian-Albrechts University of Kiel, Kiel 24118, Germany; USDA-ARS Edward T. Schafer Agricultural Research Center, Sugarbeet Research Unit, Fargo, ND, USA; Environmental Genomics, Max Planck Institute for Evolutionary Biology, Plön 24306, Germany; Environmental Genomics group, Botanical Institute, Christian-Albrechts University of Kiel, Kiel 24118, Germany

**Keywords:** fungal pathogen evolution, plant disease, beet, selective sweeps, population divergence

## Abstract

One of the most recent crop species to be domesticated is sugar beet (*Beta vulgaris* L. ssp. *vulgaris* Doell.), which was bred for high sucrose content within the last few centuries in Europe. Crop domestication can also lead to the evolution of novel pathogens, which may spread across large geographical distances with their crop host. In this study, we addressed the recent evolution of the fungal pathogen causing the disease *Cercospora* leaf spot, *Cercospora beticola*. This pathogen has become increasingly important in sugar beet and table beet production worldwide. We used genome sequences of 326 *C. beticola* isolates collected from 4 continents from 4 closely related *Beta* subspecies (3 domesticated and 1 wild). We applied population genomic analyses to identify signatures of population differentiation and host specialization in *C. beticola* populations derived from the cultivated and wild hosts. We found evidence that *C. beticola* populations in agro-ecosystems likely originate from sea beet-infecting isolates. Intriguingly, host jumps from wild to cultivated beet occurred in at least 2 independent events as evidenced by our population data of *C. beticola* from wild beet collected in the Mediterranean and the UK. We explore the occurrence of genetic variants associated with fungicide resistance and virulence and show that standing genetic variation in *C. beticola* populations from both wild and domesticated plants may serve as a reservoir of functionally important alleles. Overall, our results highlight the ability of *C. beticola* to invade the agro-ecosystem and establish new populations, demonstrating the rapid adaptation potential of the species.

**Significance**

Fungal pathogens have a detrimental impact on food production. An important concern is the continuous emergence and dispersal of new pathogens, a phenomenon that may accelerate with global climate change. Understanding the origin and processes of dispersal of crop pathogens is crucial to prevent the emergence of future pandemics, and to foresee rates of dispersal and adaptive evolution. In our study, we addressed the recent emergence of a fungal pathogen infecting crops. We included samples of the pathogen from wild plants and demonstrated that wild plant populations may act as a reservoir of new pathogens and traits that enable crop infection or confer fungicide resistance.

## Introduction

Population genetics has proven to be a valuable tool in our efforts to understand the evolutionary trajectories of economically significant plant pathogens at a global scale (Pereira et al. 2020; Thierry et al. 2022; Feurtey et al. 2023; Taliadoros and Stukenbrock 2023). These studies provide valuable insights into the adaptation of pathogen populations to local conditions and hosts, which, in turn, offer crucial information for disease management, measures for regulating the movement of plant materials, and the use of fungicides to minimize disease outbreaks. For instance, insights into the origin of pathogen species, patterns of dispersal, processes of host specialization, and mechanisms of evolution and dispersal have been achieved by sampling of pathogen populations from different geographical regions and different hosts and by detailed analyses of polymorphic markers (e.g. Ali et al. 2014; Gladieux, Condon, et al. 2018; Spanner et al. 2021).

Host-specificity refers to the ability of certain pathogens to induce disease in particular host species or lineages within a host species (Borah et al. 2018; Li et al. 2020). The successful infection of a particular host species by a pathogen relies on the ability to overcome or avoid general and specific immune responses (Thines 2019). Molecular studies have provided mechanistic insights into the molecular interactions of plants and pathogens and have highlighted certain proteins on both host and pathogen sides, which are crucial determinants of resistance and susceptibility (reviewed in e.g. Dodds et al. 2006; Dodds and Thrall 2009). On the pathogen side, so-called effectors play determining roles in successful host invasion (Lo Presti et al. 2015; Toruño et al. 2016). Effectors are molecules secreted by pathogens to manipulate the host's physiology to the benefit of the pathogen. For example, the effector “EPIC1” is a protease inhibitor secreted by the oomycete pathogen *Phytophthora infestans* and related species infecting different plant species (Dong et al. 2014). EPIC1 targets immune-related and plant-specific proteases, thereby facilitating pathogen invasion in a host-specific manner. Another example is the effector Cmu1 produced by the biotrophic pathogen *Ustilago maydis*. This effector was shown to interfere with the salicylic acid biosynthetic pathways and thereby directly influence immune signaling (Djamei et al. 2011). Many described effectors are proteins, and the genes encoding these can be predicted according to specific sequence hallmarks. Effector genes are typically cysteine-rich (increasing their stability in the extracellular space) and contain a signal peptide for secretion into the plant apoplast or translocation into the host cytoplasm (Sperschneider and Dodds 2022). Given their fundamental role during pathogen infection, most effector-encoding genes are rapidly evolving in an ongoing evolutionary arms race with host targets (Möller and Stukenbrock 2017).

Host-specific lineages have been reported in many major fungal plant pathogens such as the rice blast fungus (*Pyricularia oryzae*, syn: *Magnaporthe oryzae*), for which cereal- and grass- specialized lineages were reported to be diverging despite the gene flow between them (Gladieux, Condon, et al. 2018). Other examples include the cultivar-driven divergence of the fungal wheat pathogen *Zymoseptoria tritici* (Hartmann et al. 2018) and closely related species of the oomycete pathogen *Phytophthora* infecting distinct host species (Dong et al. 2014). In most cases, however, the underlying traits that define host-specificity are not known and can only be predicted by comparative analyses of pathogens on different hosts.

The fungus *Cercospora beticola* is the causal agent of the disease *Cercospora* leaf spot (CLS) in cultivated beets (*Beta vulgaris* spp. *vulgaris*, which includes sugar beet, fodder beet, leaf beet, and garden or table beet, as well as wild sister species of these crops, including sea beet (*B. vulgaris* L. spp. *Maritima* (L.) Arcang.) (Tehseen et al. 2023). The pathogen undergoes multiple rounds of asexual reproduction within a single growing season (Rangel et al. 2020). Between sugar beet growing seasons, the fungus primarily survives in the form of hyphal structures called pseudostromata known to be resistant to desiccation (Rangel et al. 2020). These specialized structures, consisting of fungal tissue and remnants of host tissue, are situated within the leaf substomatal cavities of leaf remains and can remain viable for up to 2 years. While a known sexual form has not yet been identified, *C. beticola* is classified as a heterothallic ascomycete fungus. This classification arises from the identification of 2 oppositive mating-type idiomorphs (*MAT1-1-1* and *MAT1-2-1*) in different isolates within populations (Groenewald et al. 2006). Previous studies found both mating types to be present at equal frequencies in certain regions of Europe and North America, providing indirect evidence for sexual reproduction (Bolton, Secor et al. 2012; Bolton et al. 2014; Vaghefi et al. 2017). Despite the wide host range of the pathogen, including on wild and domesticated plants, information about the evolutionary history of the pathogen is scarce, and it is not known if the wide host range of the fungus in fact represents different host-specialized lineages or a generally broad host range of individual isolates (Rangel et al. 2020).

Domesticated beets and one of the wild ancestors, the sea beet, have a long history associated with humans (Tehseen et al. 2023). The wild beet species *B. vulgaris* subsp. *Maritima* has been described as highly resistant to a variety of pests and pathogens and thereby as a potential source of new disease resistance traits for sugar beet (Tehseen et al. 2023). However, little is known about the diversity and biology of pathogens in *B. vulgaris* subsp. *Maritima*. Archaeological and historic evidence suggests that the wild ancestor of beets originates from the Mediterranean region (Biancardi et al. 2020). The first reports of beet cultivations came from the ancient Greeks and Romans, who were cultivating beets for their leaves. Beetroot, the part we commonly associate with beets today, was only developed during the late stages of the Roman Empire (Biancardi et al. 2020). By the 16th century, beet cultivation had spread to Northern Europe, and fodder beets were widely used for animal feed. In the 18th century, a German chemist, Andreas Marggraf, discovered that sugar could be extracted from beets, laying the foundation for sugar beet production (reviewed in Rangel et al. 2020). This marked a significant turning point, as beets became a vital source of sugar during the Napoleonic wars when sugar cane became scarce. Eventually, human migration to North America further dispersed cultivated beets during the last few centuries. Altogether, there has been a rapid diversification and a worldwide dispersal of beets associated with human history and crop use.

The global spread of plant species is often accompanied by the undesirable spread of associated pathogens (Stukenbrock et al. 2006; Sotiropoulos et al. 2022). Pathogen dispersal can lead to new adaptations allowing pathogens to establish in new environments, eventually in agricultural environments with distinct crop cultivars or management strategies, e.g. locally used fungicides (Croll and McDonald 2017). Agricultural environments may pose very strong selection pressures on pathogens, and evolutionary analyses of genetic data may reveal genetic loci that have been important for the establishment of the pathogen in certain environments. Evidence of such adaptation can sometimes be detected as “selective sweeps” in the genome. Typical signatures of selective sweeps are reduced genetic variation and increased linkage disequilibrium (LD) across a genomic region spanning the advantageous allele (Stephan 2016). A Click or tap here to enter text. number of methods are available to detect such signatures in population genomic data by integrating different signals in the data, for example, the local site frequency spectrum and LD patterns, and accounting for an underlying demography model (Pavlidis et al. 2013; Alachiotis and Pavlidis 2018). We previously demonstrated that selection for fungicide resistance has resulted in strong selective-sweep-signatures in North-American populations of *C. beticola* (Spanner et al 2021). We hypothesize that population genomic analyses may allow the further identification of functionally relevant traits that have been important for recent adaptation.

In the present study, we analyzed the genomes of 326 *C. beticola* isolates obtained from diverse beet fields and natural sea beet habitats from different geographical locations. Our objective was to infer the global population structure of *C. beticola* and identify the factors shaping this. In particular, we aimed to assess the genetic footprints of host specialization and local adaptation within the species. We utilized single nucleotide polymorphism (SNP) data to explore genetic variation in *C. beticola*. Our findings strongly support a host-driven divergence of pathogen populations. Furthermore, we scanned the genome alignment to detect signs of recent selective sweeps and observed a significant overlap between these signatures and potential virulence factors and fungicide resistance alleles. Our study reveals host-driven divergence of an important plant pathogen but also highlights the need for surveillance of pathogens on wild crop relatives.

## Results

### Generation of *C. beticola* Population Genomic Datasets

To explore the genetic structure of *C. beticola* populations and shed light to the emergence and recent history of this economically important sugar beet pathogen, we compiled a dataset encompassing whole-genome sequence data of 326 isolates from cultivated sugar beet across 4 continents (Africa, America, Central Asia, and Europe) (supplementary table S1, Supplementary Material online). The isolates from the UK were sequenced in Germany at the Max Planck Genome Center Cologne, while the rest of the isolates were sequenced in the USA at Novogene (Sacramento, California, USA) with Illumina sequencing technology. All sequencing reads were mapped to the reference genome of *C. beticola* (De Jonge et al. 2018) to call SNPs. The read coverage across the 326 genomes ranged from 11× to 32× with an average of 21×. We identified a total of 1,068,644 SNPs among the 326 isolates. A summary of the mapping statistics and SNP calling is summarized in supplementary table S1, Supplementary Material online.

### Increased Genetic Diversity in Local Mediterranean Populations of *C. beticola*

**Fig. 1.**
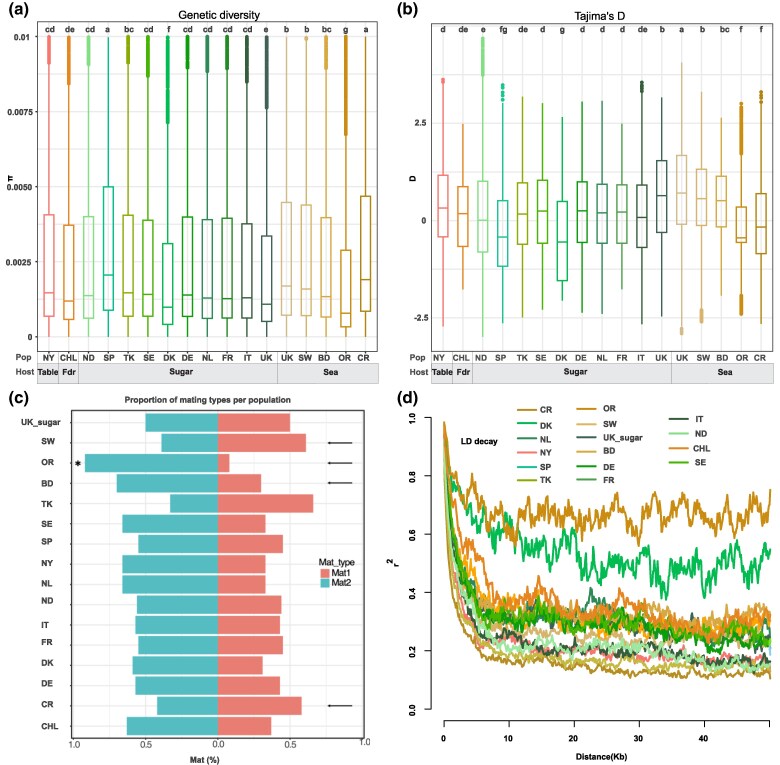
Collection of *C. beticola* isolates across continents used to infer pathogen population structure and dispersal. a) Nucleotide diversity of *C. beticola* populations in each geographic region. Kruskal–Wallis test with post-hoc pairwise Wilcoxon was used to identify significant differences (*P* < 0.05, df = 16) between the groups (see Table 1). b) Tajima's *D* of *C. beticola* populations in each geographic region. Kruskal–Wallis test with post-hoc pairwise Wilcoxon was used to identify significant differences (*P* < 0.05) between the groups (Table 1). c) Percentage of the 2 mating types occurring in each population. The asterisk indicates significant departure from a 1:1 ratio (Chi-squared test, *P*-value: 0.05, df = 1). d) LD decay for each population.

The level of standing genetic variation in populations can give insight into the recent and ancestral demographic history. To explore genetic variation in *C. beticola*, we first conducted a comparative analysis of the genetic diversity among isolates from the 16 geographical locations, revealing significant differences in median values of the parameter π, a measure of overall nucleotide diversity within populations (*P*-value < 2.2e−16, df = 16, Kruskal–Wallis test). Subsequently, we performed Wilcoxon tests to assess significant differences between pairs of populations (Fig. 1a, supplementary table S3, Supplementary Material online). Our analysis showed that the 2 Mediterranean populations, Spain and Croatia, comprise significantly higher levels of nucleotide diversity (π_Spain_ = 0.0022, π_Croatia_ = 0.0020) than every other population. We consider that the higher diversity in the Mediterranean populations could reflect that the origin of the pathogen is in Southern Europe.

Also, the populations originating from wild sea beet plants collected at the sea shore in the UK at Southwold and Bawdsey showed higher genetic diversity (mean π_Swd_ = 0.0016, π_Bawd_ = 0.0014) compared with other *C. beticola* populations. Interestingly, the UK population isolated from sea beets in Orford showed the lowest genetic diversity of all *C. beticola* populations (mean π: 0.0008), suggesting a considerable variation in diversity among geographically close populations on the wild sea beet host. The Orford population furthermore showed a negative Tajima's *D* value (Fig. 1b) reflecting an excess of low-frequency alleles possibly resulting from a population expansion after a recent bottleneck. We speculate that the pathogen population at Orford has a different demographic history compared with the other pathogen populations on UK sea beet. The different levels of genetic variation moreover indicate a “metapopulation structure” with limited gene flow between *C. beticola* populations on sea beet in the UK. Here we define “metapopulation” as genetically differentiated subpopulations with very limited gene flow but which occur in close geographical proximity.

Overall, Tajimas *D* for each of the populations was close to 0, indicating a mutation-drift equilibrium in the populations. However, some differences in Tajimas *D* values are apparent. We compared Tajima's *D* values for the other geographical *C. beticola* populations using the Kruskal–Wallis test, which revealed significant differences among pairs of populations (*P*-value < 2.2e−16, df = 16, Kruskal–Wallis test). Subsequently, we performed a pairwise Wilcoxon test to assess significant differences between the populations (Fig. 1b, supplementary table S4, Supplementary Material online). In general, sea beet-infecting populations show higher levels of Tajima's *D*, which may reflect distinct demographic processes acting on these populations. The *C. beticola* populations in Spain, Denmark, and Orford showed significantly different Tajimas *D* values (*D* < 0) compared with the rest of the populations. We speculate that an excess of low-frequency alleles (reflected by the negative Tajimas *D* values) may reflect a recent bottleneck in Denmark and Orford, as the level of nucleotide variation in these populations likewise is low. On the other hand, the high nucleotide variation in Spain and the negative Tajima's *D* value may reflect a strong population expansion.

We next addressed the mode of reproduction in the pathogen populations, considering that *C. beticola* can reproduce either asexually or by mating between individuals with compatible mating types, Mat1-1 and Mat1-2. To this end, we determined the relative frequencies of mating type idiomorphs and applied a Chi-squared test to assess statistical significance of the deviation from a 1:1 ratio, expected in a population with random mating. This analysis revealed equal mating type ratios for all populations, i.e. consistent with sexual reproduction, except for the *C. beticola* population from wild beet in Orford (Mat1-1-1:Mat1-2-1 = 3.5, Chi-squared test, *P* = 0.001, df = 1) (Fig. 1c, supplementary table S4, Supplementary Material online). In the case of the Orford population, the Mat1-1 idiomorph is not present, suggesting a primarily asexually reproducing population. Overall, however, our mating type analysis provides evidence for a prominent role of sexual reproduction in *C. beticola* populations across the different geographical regions and on the different hosts represented with our sampling.

We also addressed recombination at the genomic level. We computed the extent of LD decay for each population. LD patterns can be informative about the extent of sexual reproduction. Recently founded populations, as well as, populations that primarily reproduce asexually, will show longer blocks of LD compared with recombining populations. In line with the analysis of mating types, we found significantly longer linkage blocks in the Orford, UK and Danish populations. The LD statistic *r*^2^ was reduced to half its maximum value at 20 Kbp for the Danish population, while it never dropped to half for the Orford population (Fig. 1d, Table 1). Considering that the Danish population showed a balanced mating-type ratio, we propose that the long LD blocks derive from a recently founded, sexually reproducing population, while for the UK, Orford population, we attribute the long LD blocks to asexual reproduction.

**Table 1:** Multicontinental sampling of *C. beticola* isolates, estimates of genetic diversity, Tajima's *D*, mating types ratio and LD decay

Sampling location					π		Tajima's D				
Continent	Country	Population	Host	No of isolates	Mean	SD	Mean	St.dev	Mat1:Mat2 ratio	Private variants	Dist r2/2 (Kbp)
Asia											
	Turkey	TK	Sugar	18	0.0015	0.0035	0.1	1.22	2	4555	2.2
Europe											
	Sweden	SW	Sugar beet	12	0.0014	0.0031	0.06	1.23	0.5	2280	3.1
	Denmark	DK	Sugar beet	13	0.001	0.0023	−0.64	0.98	2.25	561	2.3
	Netherlands	NL	Sugar beet	15	0.0012	0.0032	0.05	−0.17	0.5	595	2.3
	Germany	GE	Sugar beet	14	0.0014	0.0033	0.04	1.23	0.75	1320	4.3
	France	FR	Sugar beet	11	0.0012	0.0039	0.05	1.13	0.83	725	2.9
	Spain	SP	Sugar beet	20	0.0021	0.0032	−0.38	1.25	0.83	56,980	3.1
	Italy	IT	Sugar beet	21	0.0012	0.003	0.03	1.22	1.75	657	3.9
	Croatia	CR	Sea beet	24	0.002	0.0034	−0.06	1.25	1.4	28,657	0.7
	UK	BB	Sugar beet	24	0.001	0.0032	0.52	1.25	1	417	3.2
		OR	Sea beet	14	0.0006	0.0028	−0.36	0.77	0.08	3376	-
		BD	Sea beet	10	0.0016	0.0037	0.47	1.13	0.43	1655	2.8
		SW	Sea beet	18	0.0018	0.0033	0.51	1.12	1.57	15,607	2.3
North America											
	USA	ND	Sugar beet	80	0.0013	0.0027	0.12	1.5	0.8	11,886	3.1
		NY	Table beet	24	0.0013	0.0032	0.24	1.25	0.5	7164	3
South America											
	Chile	CH	Fodder beet	8	0.0012	0.0035	0.12	1.17	0.6	157	3.9
Total				326	0.0042	0.0046	−0.34	1.49	0.99		

### The Host-driven Population Structure of *C. beticola*

**Fig. 2.**
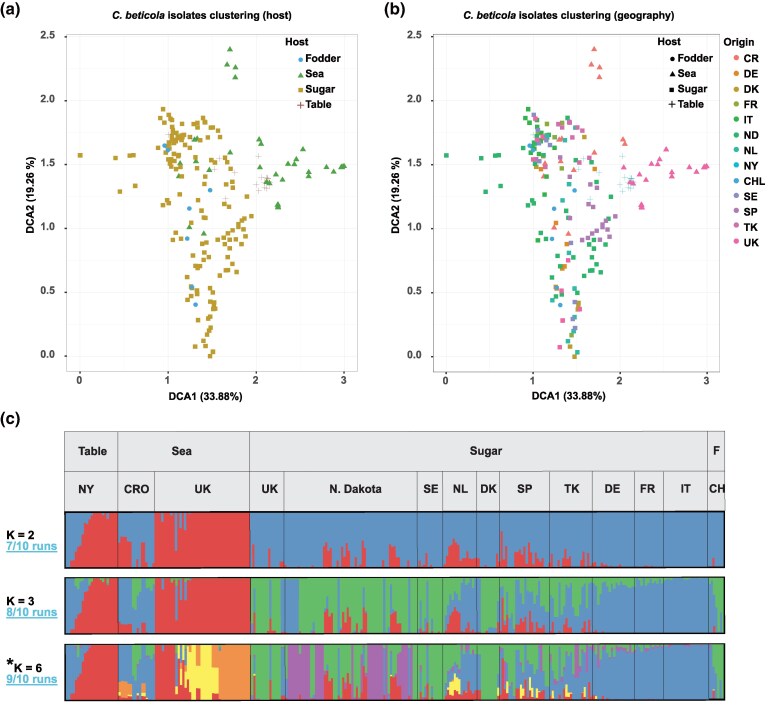
Global population structure of *C. beticola.* a, b) DCA, where the different colors reflect a) the original host of the isolate and b) the geographical origin. Shapes reflect the original host in both panels. c) The program ADMIXTURE was used to compute population structure of the species. Most fit number of hypothetical ancestral groups was identified as *K* = 6 based on the cross-validation method (see supplementary table S5, Supplementary Material online). Shown are patterns of admixture for 2, 3, and 6 hypothetical ancestral groups. Isolates originate from different hosts and countries. Codes for host plants are: Table, Table beet; Sea, sea beet; Sugar, sugar beet; F, Fodder beet. Codes for geographical origins are: NY, New York; CRO, Croatia; UK, United Kingdom; N. Dakota, North Dakota; SE, Sweden; NL, The Netherlands; DK, Denmark; SP, Spain; TK, Turkey; DE, Germany; FR, France; IT, Italy; CH, Chile.

We next characterized the population genetic structure of *C. beticola* based on a combination of methods utilizing the “independent SNP” dataset (see Materials and Methods). Firstly, we assessed the degree of clustering by implementing a detrended correspondence analysis (DCA) (Hill and Gauch 1980) (Fig. 2a and b). The DCA plot primarily reflected clustering according to the host species, and separated isolates obtained from farmlands and from the wild hosts. Interestingly, the isolates coming from sea beet and table beet clustered together. We further explored population structure using an ADMIXTURE analysis to infer the proportions of shared ancestries among the isolates (Alexander et al. 2009). We run the analysis for a number of clusters (*K*) ranging between 2 and 9 (supplementary fig. S1, Supplementary Material online). The number of clusters corresponding to the deepest subdivision was selected, using a cross-validation analysis. Models with a *K* value larger than 6 induced only a small decrease in the cross-validation error (Fig. 2b, supplementary table S5, Supplementary Material online). At *K* = 2, isolates from UK-sea beet and New York table beet shared the same ancestry, while the sugar and fodder beet-infecting isolates constituted the other cluster. At *K* = 3, the isolates were further separated within the sugar-fodder beet cluster, revealing 2 globally occurring sugar beet infecting clusters. Finally, at *K* = 6, we observed further sub-clustering within the UK sea beet-infecting isolates, mainly driven by the geographic origin of the isolates (at Bawdsey, Southwold, and Orford). In addition, at *K* = 6, the North Dakota sugar beet-infecting population was divided into 2 genetic clusters, although obtained from the same geographical location. We note that shared ancestry, in general, is extensive among the *C. beticola* isolates investigated here. In line with the analysis of genetic diversity, the Croatian population encompasses most of ADMIXTURE's hypothetical ancestral clusters, possibly reflecting that the population is older, and results from a longer history of admixture of different populations in the region.

### Host Species is a Significant Factor Separating *C. beticola* Populations

**Fig. 3.**
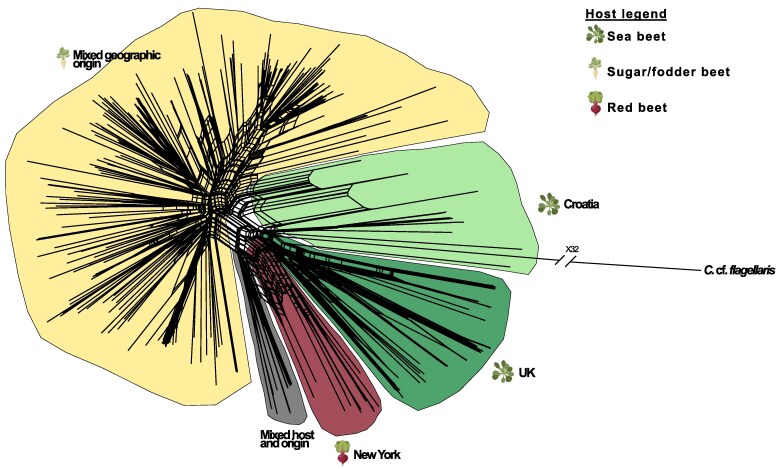
Neighbor network of *C. beticola* isolates. The network was inferred with SplitsTree v. 6. The main clusters identified by the analyses are colored according to the host from which the isolates were originally obtained (yellow = sugar beet, green = sea beet, red = Table beet). Moreover, the tree includes an admixed cluster = gray, with isolates of different geographical and host origin. Parts of the figure were obtained from BioRender.com (2020) using the terms “sugar beet”, “table beet”, and “arabidopsis” (https://app.biorender.com/biorender-templates).

Host specialization and geographic isolation are 2 factors that often play a key role in reproductive isolation in filamentous plant pathogens (Möller and Stukenbrock 2017). To illuminate the role of geographic separation and host specialization as the driving factors of *C. beticola* population divergence, we first performed 2 AMOVAs (supplementary fig. S2, Supplementary Material online, Table 2). To assess the fraction of variation associated with the geographic distance, we used all the available isolates. In this analysis, the major component of genetic variation, 77%, is explained by variation within individual populations. An additional 19% of the variation is explained by genetic variation within countries. Only 9% of the variation is explained by variation between countries. Furthermore, we found did not find evidence that geography at the continental scale explains variation between *C. beticola* populations. Overall, the AMOVA suggested that genetic variation within populations explains most of the variation in *C. beticola*, while variation between countries and continents is nonsignificant (*P*-value: 0.17 and 0.82, respectively). This observation indicates a recent dispersal of *C. beticola* and/or extensive gene flow across long distances. We further generated a neighbor network, which further supports a lack of geographical population structure and a recent differentiation of the populations (Fig. 3). The neighbor network moreover suggests that genetic variation has been homogenized by gene flow among isolates from different countries.

**Table 2:** AMOVA results for groups according to teir geographic origin and host

Factor	Source of variation	df^a^	SS^b^	% var^c^	*P*-value^d^
Geography					
	Among continents	3	72,037.32	−5.13	0.82
	Among countries within continents	10	255,615.11	8.89	0.17
	Among samples within countries	15	170,422.76	18.98	0.001
	Within samples	260	1,174,140.96	77.26	0.001
	Total	288	1,672,216.17	100	
Host					
	Among hosts	1	79,184.24	21.42	0.03
	Among samples within host	4	73,058.52	15.83	0.001
	Within samples	49	318,947.12	62.75	0.001
	Total	54	471,189.89	100	

^a^df, degrees of freedom.

^b^SS, Sum of squares.

^c^% var, percentage of variation explained.

^d^ *P*-value, obtained from 1.000 permutations.

We next tested the effect of the host on the population structure of *C. beticola*. For this analysis, we focused on the UK, the only country from which we had populations obtained from different hosts within the same region. Although, the main contribution of the variance again is explained by variation within populations, the effect of the host species (sugar vs. sea beet) was significant (AMOVA, *P*-value: 0.03, df = 1). As much as 21% of the genetic variance was associated with the host, suggesting a strong factor of host genetics in driving genetic differentiation of the UK *C. beticola* populations. We note that other factors, such as local adaptation and genetic drift, may further contribute to the population structure of the pathogen. Altogether, we conclude, however, that for the geographically coexisting *C. beticola* populations on wild and cultivated beet in the UK, there is a significant effect of host type on the partition of genetic variance.

### Phylogenomic Analysis Suggests an Ancient Split of Wild Beet-infecting *C. beticola* Populations and Recurrent Introductions to the Agro-ecosystem

**Fig. 4.**
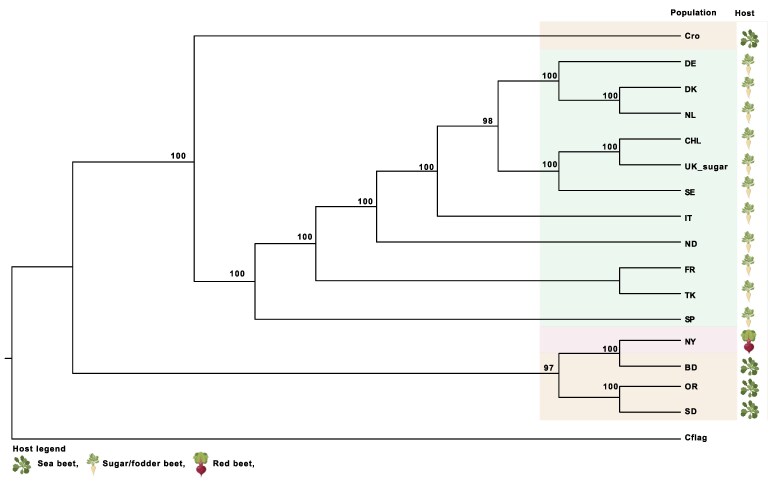
Evolutionary relationship between *C. beticola* populations. a) Phylogenetic tree using PoMo (Maximum likelihood inference, loglikelihood: −857,128.545) to assess the evolutionary relationship between *C. beticola* populations. Branch numbers reflect maximum likelihood bootstrap values. The tree was rooted using the sister species *Cercospora flagellaris*. Parts of the figure were obtained from BioRender.com (2020) using the terms “sugar beet,” “table beet,” and “arabidopsis” (https://app.biorender.com/biorender-templates).

Domesticated beet was dispersed across Europe during the medieval era and later to the Americas with European migrants (Biancardi et al. 2020). To test if early dispersal of *C. beticola* coincided with the dissemination of beet cultivation, we assessed the evolutionary relationships of the *C. beticola* populations. For this purpose, we used a maximum likelihood phylogenetic analysis, IQ-TREE, implementing a polymorphism-aware model (PoMo) (Minh et al. 2020) (Fig. 4). Using the sister species *Cercospora flagellaris* as the root for the *C. beticola* populations, the analysis revealed 2 primary population clades: a lineage comprising the Mediterranean, sugar, and fodder beet-infecting populations, and 1 lineage comprising the UK sea beet-infecting isolates and the NY Table beet-infecting isolates. An intriguing conclusion from the phylogenetic analysis is that *C. beticola* was thereby introduced at least 2 times to agricultural ecosystems. We base this conclusion on the fact that the 2 main clusters each comprise a distinct sea beet population, 1 in Southern Europe (Croatia), which may have given rise to the sugar beet-infecting *C. beticola* pathogen, and 1 in Northern Europe (the UK sea beet population), which may have given rise to the table-beet-infecting *C. beticola* population.

### Ongoing Host-driven Divergence of *C. beticola* in the UK

**Fig. 5.**
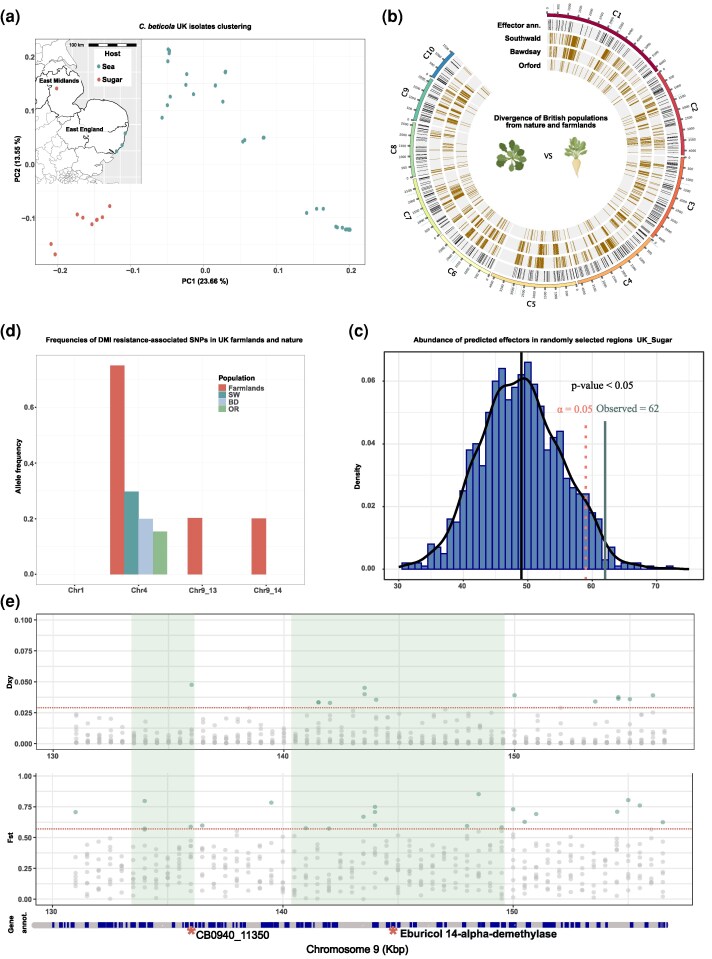
Distribution of highly diverging regions across the genome in *C. beticola* populations from wild and domesticated hosts. a) PCA based on SNP data from the UK *C. beticola* isolates. Color reflects the host from which each isolate was obtained. Inset: a map of UK with the sampling sites marked. b) Genomic map of highly diverging regions for the Southwold (SW), Bawdsey (BD), Orford (OR) populations against the UK farmland population. The first track shows the coordinates of genes encoding predicted effectors. c) To determine if genes encoding putative effectors are enriched in selective sweep regions, we performed an enrichment analysis based on the distribution of predicted effector abundance in randomly selected genomic regions of the same number and length as the selective sweep regions using 10,000 runs of random resampling of genomic regions (see Materials and Methods). d) Allele frequencies of 4 SNPs previously demonstrated to be associated with fungicide resistance in UK wild and domesticated host-infecting populations. e) D_XY_, and F_ST_ analyses between the UK populations of Southwold (sea beet infecting), and Bracebridge (sugar beet infecting) across a region of chromosome 9. Highlighted are 2 genome loci previously identified as associated with fungicide resistance (Spanner et al. 2021). At the bottom, the gene annotation presented. Genes are showed in blue. the red stars show the 2 mutations associated with fungicide resistance on chromosome 9, one located in the Eburicol 14-alpha methylase gene and the other in a gene encoding a hypothetical protein of unknown function.

Despite the fact that the UK isolates obtained from farmlands and wild plants were collected from a distance of roughly 150 km apart, our ADMIXTURE analysis suggests that they have different ancestries (Fig. 2). Furthermore, there was no evidence of admixture between these populations. Indeed, a Principal Component Analysis (PCA) including only the UK populations showed a clear separation between the sugar and sea beet-infecting populations (Fig. 5a, supplementary fig. S3, Supplementary Material online). As the AMOVA described above indicates, some genetic variation is explained by the host from which the isolates were obtained. Therefore, we hypothesized that divergent host specialization may act as a barrier to gene flow. To pinpoint the genomic components associated with host specialization in the UK, we performed genome-wide scans for highly divergent regions between the 3 sea and 1 sugar beet infecting populations sampled in the UK. We computed the population divergence statistic F_ST_ and D_XY_ for nonoverlapping windows of 5 Kbp along the genome. We considered genomic windows showing F_ST_ and D_XY_ values in the 0.05%ile to be outlier genomic regions representing loci of increased divergence between populations. To further control for the effects of varying genetic diversity that can influence the F_ST_ results, we focused on the regions that have been identified as significant by both statistics. A map of the highly diverging regions between each of the 3 sea beet and the sugar beet populations is shown in Fig. 5b.

Due to their determining role in host-pathogen interactions, genes encoding effector proteins often experience strong selective pressure (Möller and Stukenbrock 2017). Effector proteins play a crucial role in plant-pathogen interactions, as pathogen lineages with the right effector repertoire can suppress the defenses of a specific host and successfully complete their life cycle. Therefore, we conducted a permutation test to determine whether effector genes showed a significant overrepresentation in the highly diverging regions. To this end, we evaluated the proportion of predicted effector genes within the highly diverging regions in comparison to the remainder of the genome. Our results clearly indicated that effector genes are enriched in the highly diverging regions when compared with randomly selected genomic regions, with 62 predicted effector genes in these regions (Fig. 5c). The over-representation of effector genes in highly diverged regions supports our hypothesis that this group of genes is under strong selection, likely due to their prominent function in virulence. We speculate that different repertoires of immune receptors and resistance genes in beet cultivars and species drive adaptive changes in effectors of *C. beticola*.

### The Recently Described Avirulence Gene, *AvrCR4*, has Been Present at Low Frequencies in *C. beticola* Populations

Sugar beet lines harboring the major resistance gene *BvCR4* are resistant to *C. beticola* (Chen et al. 2024). This resistance gene was recently identified and introduced into cultivated sugar beet. The *BvCR4* resistance gene has, however, rapidly lost its efficacy as virulent *C. beticola* have emerged in the last few years. In a recent publication, the avirulence gene was identified and described as an effector-like small, secreted protein (Chen et al. 2024). Deletion of the avirulence gene *AvrCR4* is associated with high levels of virulence on *BvCR4* beet lines. With the available sequence information of *AvrCR4*, we scanned our geographical population samples for individuals that carried the *AvrCR4-*deletion. In line with the findings from Chen et al. 2024, we find that the deletion is very rare, and observed in only 2 out of the 289 isolates we analyzed in this study. The 2 sugar beet isolates that carry the deletion originate from the UK and Sweden. Intriguingly, our samples were collected between 2011 and 2017, before the release of the widely used *BvCR4* sugar beet variety into the market in 2020. Therefore, the recorded frequency of the deletion reflects the period before the introduction and shows that the deletion mutation was already present in *C. beticola* populations before the introduction of the *BvCR4* resistance gene.

### High Frequencies of Fungicide Resistance-associated Alleles in *C. beticola* Populations

In farmlands, there is often an extensive use of pesticides, such as fungicides applied to manage fungal disease (Stukenbrock and Mcdonald 2008). Demethylation inhibitor compounds (DMIs) are prominently used to control *C. beticola* populations in sugar beet fields worldwide. In a previous study, 4 SNP alleles were reported to be associated with DMI resistance (Spanner et al. 2021). To examine the difference in the abundance of the identified DMI resistance alleles, we recorded their frequency in the 4 UK populations, including isolates from wild and cultivated beet (Fig. 5d). The mutations associated with fungicide resistance were located at positions 2,402,041 on chromosome 1, 849,506 on chromosome 4, and 1,358,331 and 1,451,478 on chromosomes 9. All the mutations are located in hypothetical proteins except the latter, which is a synonymous mutation within the coding region of the gene encoding for eburicol 14-alpha demethylase CB0940_11379, otherwise known as the gene encoding the DMI fungicide target CbCYP51 (Bolton, Birla, et al. 2012). Three out of 4 mutations were absent in the sea beet-infecting populations, while the mutation on chromosome 4 was found in all populations at varying frequencies (15% to 33%). In contrast, the mutations on chromosome 4 and 9 were found in the UK sugar beet infecting populations in high frequencies (in 75%, 53%, and 53% of all isolates for chromosome 4, chromosome 9: 1,358,331, and 1,451,478, respectively). None of the UK populations carried the mutation on chromosome 1. Both mutations on chromosome 9 are in close vicinity of genomic regions that we found to be significantly diverged between the UK farmland and wild populations (Fig. 5e). Altogether, this analysis shows a strong selection for DMI fungicide resistance in UK farmlands, while these mutations are rare or absent in populations infecting wild beet plants. We further recorded the frequencies of DMI fungicide resistance in *C. beticola* populations worldwide. Fungicide resistance-associated variants were present in all populations around the world at different frequencies (supplementary figs. S4 to S14, Supplementary Material online), and we conclude that genetic variants associated with DMI resistance are present in both the farmland and wild populations of *C. beticola* and may be readily exchanged between isolates.

## Discussion

### Lineages of *C. beticola* Have Recently Specialized to a New Crop Species in Agro-ecosystems

Understanding the factors driving the evolutionary trajectories of crop pathogens is essential to designing surveillance strategies and preventing future epidemics. Here, we analyzed a population genomic dataset of the globally present pathogen of sugar beet, *C. beticola*, to investigate patterns of genomic variation and signatures of divergent host specialization. To this end, we used an extensive global sampling of the pathogen from wild and domesticated hosts to assess the population structure and the extent of host specialization on wild and domesticated plants. Our analyses were designed to test the hypothesis that agricultural practices and the domestication of beets, involving substantial genetic changes and phenological differences between wild and cultivated plants, have shaped the recent evolution of *C. beticola* lineages. Our detailed population genomic analyses provide evidence that *C. beticola* is subdivided into multiple lineages associated with wild and domesticated hosts. Our inference of ancestry contribution revealed 6 main ancestral clusters. Intriguingly, isolates from the wild showed different ancestries than those from farmlands. This separation was even more surprising for isolates collected at a relatively small distance (within 200 km) in UK. The UK isolates from farmlands and wild sea beet showed different ancestries, and there was no evidence of extensive gene flow between the populations. This indicates some extent of host specialization among closely related lineages of *C. beticola*. Host specialized lineages were also reported for major plant pathogens like the rice blast fungus (*Pyricularia oryzae,* syn: *Magnaporthe oryzae*) (Gladieux, Condon, et al. 2018) and the wheat pathogen *Zymoseptoria tritici* (Hartmann et al. 2018). In Hartmann et al. (2018), the authors used statistical methods to identify genes under selection in different host-specific lineages of the pathogens. Signatures of positive selection were identified in known effector genes, indicating adaptation to distinct wheat cultivars. Moreover, genes associated with fungicide resistance were found to co-localize with selective sweep regions in the wheat pathogenic fungus *Z. tritici* (Hartmann et al. 2018). Highly divergent genomic regions between *C. beticola* populations from the wild and from farmlands were also found here to be enriched with predicted effector genes.

Further evidence of host-specific divergence was obtained from an AMOVA. AMOVA results showed that the effect of geographic distance is not significant on the partition of genetic variance of the species. That is in line with previous works that assessed the population structure of *C. beticola* isolates from farmlands and concluded the presence of a shallow population differentiation (Vaghefi, Kikkert, et al. 2017; Vaghefi, Nelson, et al. 2017; Knight et al. 2019). Our analysis included isolates of *C. beticola* from the wild ancestor of beet, the sea beet. This allowed us to assess the significance of the host in the species population structure and estimate the proportion of genetic variance associated with it. The analysis showed that the host significantly explained 21.42% of the variation between *C. beticola* populations and supported the hypothesis that the different hosts drive genetic differentiation in pathogen populations.

### Sugar Beet Infecting Populations of *C. beticola* Have Likely Emerged in the Mediterranean Region with Subsequent Introgressions From Wild Beet Species

A primary indicator of the center of origin of a species is the high genetic diversity compared with other locations where the species was later introduced. In the case of *C. beticola*, we observed higher diversity primarily in the Mediterranean region and secondarily in the wild sea beet population collected in the UK. Some studies have highlighted the effect of host domestication in the emergence of its associated pathogens, including the wheat-infecting pathogen *Z. tritici* responsible for the *Septoria tritici* blotch (Stukenbrock et al. 2007), the rice blast fungus *Magnaporthe oryzae* (Couch et al. 2005), the corn smut fungus *Ustilago maydis* (Schweizer et al. 2021), and the barley net blotch fungus, *Pyrenophora teres* (Taliadoros et al. 2024). The Mediterranean Sea was previously proposed as the centers of diversity and origin of cultivated beets (Biancardi et al. 2020). An elevated diversity in that region indicates that the populations are older and may represent a center of origin. We speculate that the emergence of *C. beticola* could have coincided with the early domestication of the beet host in Southern Europe.

In our study, we also assessed the evolutionary relationships between the populations using polymorphism-aware phylogenetic models. We found a common origin of the sugar beet and fodder beet infecting populations that formed a clade with the Croatian wild population, which was early diverging within the clade. This suggests a Mediterranean origin of the sugar/fodder beet populations. In the second clade, the UK wild and NY table beet populations were clustered, which suggests that the UK origin of the NY population has a different origin than that of the other pathogen populations included in this study. The different origins of the sugar beet-infecting and table beet-infecting populations lead to an intriguing conclusion that *C. beticola* was introduced to the agro-ecosystem on multiple occasions. Based on the widespread occurrence of ancestral variation, we speculate that the migration history of *C. beticola* reflects a recent range expansion of different ancestral genotypes giving rise to present-day admixed populations.

Our extended *C*. *beticola* dataset allowed us to conduct a detailed exploration of the demographic history of the species and assess the impact of host domestication, which should be a future step to further understand the emergence and spread of the CLS disease.

### Sexual Reproduction Contributes to Evolution of *C. beticola* Populations

We used SNP data to calculate genetic diversity within *C*. *beticola* populations. Interestingly, *C*. *beticola* exhibited a higher level of nucleotide diversity (π) in comparison to well-studied crop pathogens like the wheat pathogen *Z. tritici* (Hartmann et al. 2018), the wheat powdery mildew pathogen *B. graminis f.sp. tritici* (Sotiropoulos et al. 2022), and the rice blast fungus *M. oryzae* (Zhong et al. 2018). Genetic variation plays a crucial role in the ability of a pathogen to rapidly adapt; therefore, the elevated genetic diversity in *C*. *beticola* may be a significant factor contributing to the pathogen's successful survival and dispersal. Disparities between species may be attributed to variations in the frequency of sexual recombination, historical demographic events, such as bottlenecks, and gene flow. Our analyses of LD and the mating-type frequency distribution support the hypothesis that *C*. *beticola* frequently undergoes sexual recombination. This is in line with previous reports for North American and European populations (Vaghefi, Nelson, et al. 2017; Knight et al. 2019). An exception is observed in the UK population from Orford, where clonality appears to be more prevalent. Alternatively, the Orford population represents a very young founder population in which genetic diversity is still to be built up. The population genetic structure of the UK population reveals the presence of 4 distinct genetic clusters, 3 obtained from natural environments and 1 from sugar beet fields. Despite these isolates coexisting in a relatively small region, they belong to distinct clusters with limited signs of gene flow between them. This observation suggests potential host specialization, reflecting a meta-population structure and constraints on gene flow, particularly between isolates adapted to farmlands and to wild plants. A recent study of *Z. tritici* in Iran likewise found evidence for a metapopulation structure where populations of the pathogen infecting different hosts were genetically isolated (Fagundes et al. 2024).

### Host Species and Agricultural Practices Drive Adaptive Evolution in *C. beticola*

To identify evidence of local adaptation, we utilized a variety of methods to identify genomic regions that are highly diverging between the 1 farmland and the 3 wild populations collected in the UK. In total, we identified 214 highly diverging regions between these populations. Diverging genomic regions between farmland and wild *C. beticola* populations could harbor important host-specificity loci, and we indeed find that these were enriched with predicted effector genes, further highlighting the importance of the host as a main driver of rapid evolution in this pathogen.

A recent study reported genomic loci associated with DMI resistance (Spanner et al. 2021). We recorded the abundance and frequency of DMI resistance alleles in the 4 UK populations. We found 3 of 4 variants associated with fungicide resistance present in high frequencies in the UK farmland population. In contrast, we found only 1 of the 4 variants in low to moderate frequencies present in the wild populations. Furthermore, 2 of the DMI resistance loci are located in the highly diverging genomic regions, possibly reflecting a rapid adaptation of the pathogen to the agro-ecosystem. DMI compounds are used to manage *C. beticola* populations in all sugar beet growing regions, including the UK (Liebe et al. 2023). Furthermore, we found fungicide-resistant associated alleles in high frequencies in various populations around the world.

Our data does not allow us to identify the origin and source of genetic variants linked to DMI resistance. However, we consider 2 likely scenarios: (i) The mutations may have already been present in the wild as part of natural genetic variation and subsequently increased in frequency in farmland populations in response to the selective pressures imposed by field treatments. (ii) Alternatively, these could be novel mutations that evolved within farmland populations of *C. beticola* and subsequently introgressed into populations of the pathogen on wild hosts. Under both scenarios, *C. beticola* populations on wild beet species may act as a reservoir of virulence or fungicide resistance-related alleles. Further research is needed to unravel the emergence and spread of DMI resistance in *C. beticola* and the relevance of genetic exchange between populations on wild and cultivated hosts. Our study underlines the importance of studying pathogen populations on wild plants in order to understand the rapid evolution of crop pathogens. Close relatives of crop pathogens that infect wild plant, may be sources of genetic variants that confer new virulence specificities or fungicide resistances, as demonstrated for the *C. beticola* pathogen.

## Conclusion

Using a population genomics framework, we show that *C. beticola* is subdivided into multiple lineages associated with cultivated and wild hosts. We further present evidence of limited genetic exchanges between them. Our genetic diversity and phylogenetic analyses provide evidence of a parallel demographic history between the host and the pathogen and multiple introductions of the pathogen to the agro-ecosystem. This adds to the growing body of work highlighting the important role of human activities, such as domestication, migration, and trade in the emergence and spread of plant pathogens. Furthermore, our results enhance our understanding of the evolutionary forces driving the diversification of *C. beticola* and underscore the importance of research on wild plant pathogens as well as the utility of genomic data for epidemiological surveillance of fungal plant pathogens.

## Materials and Methods

### Genome Data

Out of 326 *C. beticola* whole genomes were sequenced using Illumina technology. The sequenced isolates were sampled from cultivated and wild hosts on 4 different continents (Table 1 and supplementary S1, Supplementary Material online). Eighty isolates were collected from North Dakota State University experimental sugar beet fields in Fargo and Foxhome, North Dakota, USA. Twenty-four isolates were collected from 2 table beet fields in New York, USA. Seven isolates were collected from a fodder beet field in Chile. Twenty-two isolates were collected from a sugar beet field, in the UK. Furthermore, 12 isolates were collected from a sugar beet field in Sweden. Isolates were also collected from single sugar beet fields in 6 continental European countries. Specifically, 12 isolates were collected from a field in Denmark, 18 from Germany, 11 from France, and 19 from Spain. Additionally, 21 isolates were collected from 5 fields in Italy. Moreover, we included 18 isolates from Turkey. To capture the natural variation of the species in continental and Northern Europe, we included 23 isolates from 2 natural habitats of sea beets in Croatia and 43 isolates from 3 natural sea beet locations in the UK (Southwold, Bawdsey, and Orford) (Table 1).

### Read Mapping and Variant Calling

To extract high-quality SNPs, we processed and aligned Illumina sequencing reads to a reference genome. Initially, the sequencing adapters and low-quality nucleotide bases were filtered using the software Trimomatic version 0.38 (Bolger et al. 2014), applying a PHRED quality score of 33 and a minimum read length of 30 base pairs (bp). To control for potential overrepresentation artifacts, overlapping reads were merged with PEAR version 0.9.11 (Zhang et al. 2014).

Subsequent alignment of individual reads was performed using the Cb09-40 *C. beticola* reference genome (De Jonge et al. 2018). To this end, we used the Burrows-Wheeler Aligner version 0.7.17 (Li and Durbin 2010) and Stampy v. 1.0.20 (Lunter and Goodson 2011) (see supplementary table S2, Supplementary Material online for details). SNP calling was performed using the GATK HaplotypeCaller version 4.2.18 (McKenna et al. 2010), generating a variant call format (VCF) file containing raw SNP calls. Next, to remove low-quality SNPs, we followed a “hard-filtering” approach in accordance with GATK's best practice guidelines. Specifically, the following filtering criteria were applied: (i) the ratio of SNP call quality to sequencing depth had to be higher than 2, (ii) a minimum sequencing depth of 8 was required, (iii) SNPs had to have a mapping quality score >40, (iv) the allele-specific rank sum test for mapping qualities of reference (REF) versus alternate alleles had to be >−12.5, (v) the allele-specific rank sum test had to be higher than −8, and (6) each genome required an average read coverage of at least 2. The filtering process was performed using GATK VariantFiltration version 4.0.11 (McKenna et al. 2010).

After the hard-filtering process, a final dataset of 1,129,645 variants was obtained, 893,266 of which were biallelic SNPs. This dataset is referred to as the “full high-quality dataset.”

Many clustering analyses assume that the genetic markers are independent. To meet this assumption, the “full high-quality dataset” was further filtered based on patterns of LD decay, ensuring a minimum distance of 2 kilobase pairs (Kbp) between SNPs. This threshold was determined by calculating the decay of LD (*r*²/2) averaged across populations (Table 1). Following LD-based filtering, a final dataset comprising 44,229 biallelic SNPs was retained. This dataset is referred to as the “independent SNP dataset.”

### Genetic Diversity, Neutrality Tests, and LD

We used the “full high-quality dataset” to compute and compare genetic variation among populations. The software pixy (Korunes and Samuk 2021) was applied to obtain the genetic diversity estimator π and the population divergence statistics F_ST_ and D_XY_ in nonoverlapping windows of 10 Kbp. The tool PopLDdecay (Zhang et al. 2019) with default parameters calculates LD decay. Tajima's *D* was estimated with VCFtools v. 0.1.17 (Danecek et al. 2011). Significant differences in the level of genetic diversity and Tajima's *D* values were assessed with the nonparametric Kruskal–Wallis test and the pairwise Wilcoxon with Bonferroni correction. The selection of these nonparametric tests, rather than a parametric test (e.q. ANOVA), was made because not all group data followed a normal distribution and the groups did not have equal variances, as parametric tests assume.

### Population Genetic Structure

We inferred population genetic structure using 3 different approaches: we conducted a DCA (Hill and Gauch 1980), we applied an analysis of admixture using ADMIXTURE version 1.3 (Alexander et al. 2009), and we performed a Neigbor-Net analysis using SplitsTree v 6 (Huson and Bryant 2024). These analyses were based on the “independent SNP dataset.” DCA analysis was performed using the R package vegan v. 2.6.2 and DCA visualization with R package ggplot2 (Wickham 2016). We further characterized population structure using the software ADMIXTURE. We first ran ADMIXTURE assuming *K*-values between 1 and 10. The most fit *K* value was assessed based on the cross-validation error (Alexander et al. 2009) (supplementary table S5, Supplementary Material online). Finally, to address the effect of geographic distance and individual host on the population structure, we employed an analysis of molecular variance (AMOVA) (Excoffier et al. 1992) and performed this on the same dataset. To this end, the isolates were grouped geographically according to the country and the continent of their origin, and according to the host they were isolated from. The R package poppr (Kamvar et al. 2015) was used to perform the AMOVA. Since our dataset encompass isolates from different hosts collected in the UK, we could specifically assess the significance of geographical origin and host specialization in 2 independent analyses. To assess the significance of geography, we used data from all available *C. beticola* isolates, while for the analyses of the host specialization; we focused on the UK *C. beticola* populations from sea beet and sugar beet.

### Mating Types

*MAT1-1 and MAT1-2* mating-type sequences were obtained from GenBank (accession numbers DQ264736.1 and JN863091.1, respectively) (Groenewald et al. 2006; Bolton, Secor et al. 2012). SPAdes (Bankevich et al. 2012) with default parameters was run to create complete de novo assemblies of *C. beticola* genomes. Subsequently, BLASTN with default parameters (Camacho et al. 2009) was used to assess the mating type of each isolate.

### Evolutionary Relationships Using PoMo

We employed the PoMo, as implemented in the IQ-TREE software (Minh et al. 2020) to reconstruct relationships among *C. beticola* populations. To convert our FASTA file to the count file format, we utilized the FastaVCFtoCount.py script provided with the PoMo software. We selected a substitution model based on the Bayesian Information Criterion. To this end, the ModelFinder algorithm implemented in the IQtree version 2.0.3 was used. To obtain branch support values, we perform 1000 bootstrap replicates, employing IQ-TREE bootstrap approximation option. The evolutionary relationship of the *C. beticola* populations was reconstructed with the sister species *C. flagellaris* as an outgroup.

### Genome Scans for Population Divergence

The software pixy was used to estimate the population divergence statistics F_ST_ and D_XY_ in nonoverlapping windows of 10 Kbp. Genes encoding putative secreted proteins were identified as proteins exhibiting a signal peptide using the program SignalP (Almagro Armenteros et al. 2019). SignalP-5 predicts the presence of a signal peptide and the location of its cleavage sites. TMHMM v. 2.0 was used for the prediction of transmembrane proteins (Krogh et al. 2001), which were excluded for the further analyses. The remaining genes were used to predict putative apoplastic and cytoplastic effectors with the deep learning tool EffectorP v.3 (Sperschneider and Dodds 2022).

Next, the prevalence of predicted effector genes in the diverging regions compared with other genomic regions was examined using a permutation test (custom script available on GitHub: https://github.com/Jimi92/). In brief, the abundance of predicted effectors was counted in regions of the same size and number equal to the size distribution of the diverging regions. As many as 10,000 replicate runs of random resampled regions were performed. Genome-wide maps of the diverging regions were created using Circos v. 0.69-9 (Krzywinski et al. 2009).

## Supplementary Material

evaf053_Supplementary_Data

## Data Availability

Raw reads for the *C. beticola* isolates sequenced for this work are available in the NCBI short- read archive under BioProject PRJNA1174990. Moreover, information files, custom scripts and workflows are available at the Github repository: https://github.com/Jimi92/Cercospora_beticola_pop_gen.

## References

[evaf053-B1] Alachiotis N, Pavlidis P. RAiSD detects positive selection based on multiple signatures of a selective sweep and SNP vectors. Commun Biol. 2018:1(1):79. 10.1038/s42003-018-0085-8. 30271960 PMC6123745

[evaf053-B2] Alexander DH, Novembre J, Lange K. Fast model-based estimation of ancestry in unrelated individuals. Genome Res. 2009:19(9):1655–1664. 10.1101/gr.094052.109. 19648217 PMC2752134

[evaf053-B3] Ali S, Gladieux P, Leconte M, Gautier A, Justesen AF, Hovmøller MS, Enjalbert J, de Vallavieille-Pope C. Origin, migration routes and worldwide population genetic structure of the wheat yellow rust pathogen *Puccinia striiformis* f.sp. *tritici*. PLoS Pathog. 2014:10(1): e1003903. 10.1371/journal.ppat.1003903. 24465211 PMC3900651

[evaf053-B4] Almagro Armenteros JJ, Tsirigos KD, Sønderby CK, Petersen TN, Winther O, Brunak S, von Heijne G, Nielsen H. Signalp 5. 0 improves signal peptide predictions using deep neural networks. Nat Biotechnol. 2019:37(4):420–423. 10.1038/s41587-019-0036-z. 30778233

[evaf053-B5] Bankevich A, Nurk S, Antipov D, Gurevich AA, Dvorkin M, Kulikov SA, Lesin VM, Nikolenko SI, Pham S, Prjibelski AD, et al SPAdes: a new genome assembly algorithm and its applications to single-cell sequencing. J Comput Biol. 2012:19(5):455–477. 10.1089/cmb.2012.0021. 22506599 PMC3342519

[evaf053-B6] Biancardi E, Panella LW, Mcgrath J, Mitchell. The origin of beets second edition. Berlin: Springer; 2020.

[evaf053-B7] Bolger AM, Lohse M, Usadel B. Trimmomatic: a flexible trimmer for illumina sequence data. Bioinformatics. 2014:30(15):2114–2120. 10.1093/bioinformatics/btu170. 24695404 PMC4103590

[evaf053-B8] Bolton MD, Birla K, Rivera-Varas V, Rudolph KD, Secor GA. Characterization of CbCyp51 from field isolates of *Cercospora beticola*. Phytopathology. 2012:102(3):298–305. 10.1094/PHYTO-07-11-0212. 22085297

[evaf053-B9] Bolton MD, de Jonge R, Inderbitzin P, Liu Z, Birla K, Van de Peer Y, Subbarao KV, Thomma BPHJ, Secor GA. The heterothallic sugarbeet pathogen *Cercospora beticola* contains exon fragments of both MAT genes that are homogenized by concerted evolution. Fungal Genet Biol. 2014:62:43–54. 10.1016/j.fgb.2013.10.011. 24216224

[evaf053-B10] Bolton MD, Secor GA, Rivera V, Weiland JJ, Rudolph K, Birla K, Rengifo J, Campbell LG. Evaluation of the potential for sexual reproduction in field populations of *Cercospora beticola* from USA. Fungal Biol. 2012:116(4):511–521. 10.1016/j.funbio.2012.01.011. 22483049

[evaf053-B11] Borah N, Albarouki E, Schirawski J. Comparative methods for molecular determination of host-specificity factors in plant-pathogenic fungi. Int J Mol Sci. 2018:19(3):863. 10.3390/ijms19030863. 29543717 PMC5877724

[evaf053-B12] Camacho C, Coulouris G, Avagyan V, Ma N, Papadopoulos J, Bealer K, Madden TL. BLAST+: architecture and applications. BMC Bioinformatics. 2009:10(1):421. 10.1186/1471-2105-10-421. 20003500 PMC2803857

[evaf053-B13] Chen C, Keunecke H, Bemm F, Gyetvai G, Neu E, Kopisch-Obuch FJ, McDonald BA, Stapley J. GWAS reveals a rapidly evolving candidate avirulence effector in the *Cercospora* leaf spot pathogen. Mol Plant Pathol. 2024:25(1):e13407. 10.1111/mpp.13407. 38009399 PMC10799204

[evaf053-B14] Couch BC, Fudal I, Lebrun MH, Tharreau D, Valent B, van Kim P, Nottéghem JL, Kohn LM. Origins of host-specific populations of the blast pathogen *Magnaporthe oryzae* in crop domestication with subsequent expansion of pandemic clones on rice and weeds of rice. Genetics. 2005:170(2):613–630. 10.1534/genetics.105.041780. 15802503 PMC1450392

[evaf053-B15] Croll D, McDonald BA. The genetic basis of local adaptation for pathogenic fungi in agricultural ecosystems. Mol Ecol. 2017:26(7):2027–2040. 10.1111/mec.13870. 27696587

[evaf053-B16] Danecek P, Auton A, Abecasis G, Albers CA, Banks E, DePristo MA, Handsaker RE, Lunter G, Marth GT, Sherry ST, et al The variant call format and VCFtools. Bioinformatics. 2011:27(15):2156–2158. 10.1093/bioinformatics/btr330. 21653522 PMC3137218

[evaf053-B17] De Jonge R, Ebert MK, Huitt-Roehl CR, Pal P, Suttle JC, Spanner RE, Neubauer JD, Jurick WM 2nd, Stott KA, Secor GA, et al Gene cluster conservation provides insight into *Cercosporin* biosynthesis and extends production to the genus Colletotrichum. Proc Natl Acad Sci U S A. 2018:115(24):E5459–E5466. 10.1073/pnas.1712798115. 29844193 PMC6004482

[evaf053-B18] Djamei A, Schipper K, Rabe F, Ghosh A, Vincon V, Kahnt J, Osorio S, Tohge T, Fernie AR, Feussner I, et al Metabolic priming by a secreted fungal effector. Nature. 2011:478(7369):395–398. 10.1038/nature10454. 21976020

[evaf053-B19] Dodds P, Lawrence GJ, Catanzariti AM, Teh T, Wang CI, Ayliffe MA, Kobe B, Ellis JG. Direct protein interaction underlies gene-for-gene specificity and coevolution of the flax resistance genes and flax rust avirulence genes. Proc Natl Acad Sci U S A. 2006:103(23):8888–8893. 10.1073/pnas.0602577103. 16731621 PMC1482673

[evaf053-B20] Dodds P, Thrall P. Recognition events and host-pathogen co-evolution in gene-for-gene resistance to flax rust. Funct Plant Biol. 2009:36(5):395–408. 10.1071/FP08320. 21760756 PMC3134234

[evaf053-B21] Dong S, Stam R, Cano LM, Song J, Sklenar J, Yoshida K, Bozkurt TO, Oliva R, Liu Z, Tian M, et al Effector specialization in a lineage of the Irish potato famine pathogen. Science. 2014:343(6170):552–555. 10.1126/science.1246300. 24482481

[evaf053-B22] Excoffier L, Smouse PE, Quattro JM. Analysis of molecular variance inferred from metric distances among DNA haplotypes: application to human mitochondrial DNA restriction data. Genetics. 1992:131(2):479–491. 10.1093/genetics/131.2.479. 1644282 PMC1205020

[evaf053-B23] Fagundes WC, Hansen R, Rojas Barrera IC, Caliebe F, Feurtey A, Haueisen J, Salimi F, Alizadeh A, Stukenbrock EH. Host specialization defines the emergence of new fungal plant pathogen populations. bioRxiv. 2024. 10.1101/2024.09.30.615799, preprint: not peer reviewed.

[evaf053-B24] Feurtey A, Lorrain C, McDonald MC, Milgate A, Solomon PS, Warren R, Puccetti G, Scalliet G, Torriani SFF, Gout L, et al A thousand-genome panel retraces the global spread and adaptation of a major fungal crop pathogen. Nat Commun. 2023:14(1). 10.1038/s41467-023-36674-y PMC9958100 36828814

[evaf053-B25] Gladieux P, Condon B, Ravel S, Soanes D, Maciel JLN, Nhani A Jr, Chen L, Terauchi R, Lebrun MH, Tharreau D, et al Gene flow between divergent cereal- and grass-specific lineages of the rice blast fungus *Magnaporthe oryzae*. mBio. 2018:9(1):e01219-17. 10.1128/mBio.01219-17. 29487238 PMC5829825

[evaf053-B27] Groenewald M, Groenewald JZ, Harrington TC, Abeln ECA, Crous PW. Mating type gene analysis in apparently asexual *Cercospora* species is suggestive of cryptic sex. Fungal Genet Biol. 2006:43(12):813–825. 10.1016/j.fgb.2006.05.008. 16839791

[evaf053-B28] Hartmann FE, McDonald BA, Croll D. Genome-wide evidence for divergent selection between populations of a major agricultural pathogen. Mol Ecol. 2018:27(12):2725–2741. 10.1111/mec.14711. 29729657 PMC6032900

[evaf053-B29] Hill MO, Gauch HG. Detrended correspondence analysis: an improved ordination technique. Vegetatio. 1980:42(1-3):47–58. 10.1007/BF00048870.

[evaf053-B30] Huson DH, Bryant D. The SplitsTree app: interactive analysis and visualization using phylogenetic trees and networks. Nat Methods. 2024:21(10): 1773–1774. 10.1038/s41592-024-02406-3. 39223398

[evaf053-B31] Kamvar ZN, Brooks JC, Grünwald NJ. Novel R tools for analysis of genome-wide population genetic data with emphasis on clonality. Front Genet. 2015:6:208. 10.3389/fgene.2015.00208. 26113860 PMC4462096

[evaf053-B32] Knight NL, Vaghefi N, Kikkert JR, Bolton MD, Secor GA, Rivera VV, Hanson LE, Nelson SC, Pethybridge SJ. Genetic diversity and structure in regional *Cercospora beticola* populations from Beta vulgaris subsp. Vulgaris suggest two clusters of separate origin. Phytopathology. 2019:109(7):1280–1292. 10.1094/PHYTO-07-18-0264-R. 30785376

[evaf053-B33] Korunes KL, Samuk K. Pixy: unbiased estimation of nucleotide diversity and divergence in the presence of missing data. Mol Ecol Resour. 2021:21(4):1359–1368. 10.1111/1755-0998.13326. 33453139 PMC8044049

[evaf053-B34] Krogh A, Larsson È, Von Heijne G, Sonnhammer ELL. Predicting transmembrane protein topology with a hidden Markov model: application to complete genomes. J Mol Biol. 2001:305(3):567–580. 10.1006/jmbi.2000.4315. 11152613

[evaf053-B35] Krzywinski M, Schein J, Birol I, Connors J, Gascoyne R, Horsman D, Jones SJ, Marra MA. Circos: an information aesthetic for comparative genomics. Genome Res. 2009:19(9):1639–1645. 10.1101/gr.092759.109. 19541911 PMC2752132

[evaf053-B36] Li H, Durbin R. Fast and accurate long-read alignment with Burrows-Wheeler transform. Bioinformatics. 2010:26(5):589–595. 10.1093/bioinformatics/btp698. 20080505 PMC2828108

[evaf053-B37] Li J, Cornelissen B, Rep M. Host-specificity factors in plant pathogenic fungi. Fungal Genet Biol. 2020:144:103447. 10.1016/j.fgb.2020.103447. 32827756

[evaf053-B38] Liebe S, Imbusch F, Erven T, Varrelmann M. Timing of fungicide application against *Cercospora* leaf spot disease based on aerial spore dispersal of *Cercospora beticola* in sugar beet. J Plant Dis Prot. 2023:130(2):315–324. 10.1007/s41348-023-00708-w.

[evaf053-B39] Lo Presti L, Lanver D, Schweizer G, Tanaka S, Liang L, Tollot M, Zuccaro A, Reissmann S, Kahmann R. Fungal effectors and plant susceptibility. Annu Rev Plant Biol. 2015:66(1):513–545. 10.1146/annurev-arplant-043014-114623. 25923844

[evaf053-B40] Lunter G, Goodson M. Stampy: a statistical algorithm for sensitive and fast mapping of illumina sequence reads. Genome Res. 2011:21(6):936–939. 10.1101/gr.111120.110. 20980556 PMC3106326

[evaf053-B41] McKenna A, Hanna M, Banks E, Sivachenko A, Cibulskis K, Kernytsky A, Garimella K, Altshuler D, Gabriel S, Daly M, et al The genome analysis toolkit: a MapReduce framework for analyzing next-generation DNA sequencing data. Genome Res. 2010:20(9):1297–1303. 10.1101/gr.107524.110. 20644199 PMC2928508

[evaf053-B42] Minh BQ, Schmidt HA, Chernomor O, Schrempf D, Woodhams MD, von Haeseler A, Lanfear R. IQ-TREE 2: new models and efficient methods for phylogenetic inference in the genomic era. Mol Biol Evol. 2020:37(5):1530–1534. 32011700 10.1093/molbev/msaa015 PMC7182206

[evaf053-B43] Möller M, Stukenbrock EH. Evolution and genome architecture in fungal plant pathogens. Nat Rev Microbiol. 2017:15(12):756–771. 10.1038/nrmicro.2017.76. 29123226

[evaf053-B44] Pavlidis P, Živkovic D, Stamatakis A, Alachiotis N. Sweed: likelihood-based detection of selective sweeps in thousands of genomes. Mol Biol Evol. 2013:30(9):2224–2234. 10.1093/molbev/mst112. 23777627 PMC3748355

[evaf053-B45] Pereira D, McDonald BA, Croll D. The genetic architecture of emerging fungicide resistance in populations of a global wheat pathogen. Genome Biol Evol. 2020:12(12):2231–2244. 10.1093/gbe/evaa203. 32986802 PMC7846115

[evaf053-B46] Rangel LI, Spanner RE, Ebert MK, Pethybridge SJ, Stukenbrock EH, de Jonge R, Secor GA, Bolton MD. *Cercospora beticola*: the intoxicating lifestyle of the leaf spot pathogen of sugar beet. Mol Plant Pathol. 2020:21(8):1020–1041. 10.1111/mpp.12962. 32681599 PMC7368123

[evaf053-B47] Schweizer G, Haider MB, Barroso GV, Rössel N, Münch K, Kahmann R, Dutheil JY. Population genomics of the maize pathogen *Ustilago maydis*: demographic history and role of virulence clusters in adaptation. Genome Biol Evol. 2021:13(5):evab073. 10.1093/gbe/evab073. 33837781 PMC8120014

[evaf053-B48] Sotiropoulos AG, Arango-Isaza E, Ban T, Barbieri C, Bourras S, Cowger C, Czembor PC, Ben-David R, Dinoor A, Ellwood SR, et al Global genomic analyses of wheat powdery mildew reveal association of pathogen spread with historical human migration and trade. Nat Commun. 2022:13(1):4315. 10.1038/s41467-022-31975-0. 35882860 PMC9315327

[evaf053-B49] Spanner R, Taliadoros D, Richards J, Rivera-Varas V, Neubauer J, Natwick M, Hamilton O, Vaghefi N, Pethybridge S, Secor GA. Genome-wide association and selective sweep studies reveal the complex genetic architecture of DMI fungicide resistance in *Cercospora beticola*. Genome Biol Evol. 2021:13(9):evab209. 10.1093/gbe/evab209. 34499119 PMC8459168

[evaf053-B50] Sperschneider J, Dodds PN. Effectorp 3.0: prediction of apoplastic and cytoplasmic effectors in fungi and oomycetes. Mol Plant Microbe Interact. 2022:35(2):146–156. 10.1094/MPMI-08-21-0201-R. 34698534

[evaf053-B51] Stephan W . Signatures of positive selection: from selective sweeps at individual loci to subtle allele frequency changes in polygenic adaptation. Mol Ecol. 2016:25(1):79–88. 10.1111/mec.13288. 26108992

[evaf053-B52] Stukenbrock EH, Banke S, Javan-Nikkhah M, McDonald BA. Origin and domestication of the fungal wheat pathogen *Mycosphaerella graminicola* via sympatric speciation. Mol Biol Evol. 2007:24(2):398–411. 10.1093/molbev/msl169. 17095534

[evaf053-B53] Stukenbrock EH, Banke S, McDonald BA. Global migration patterns in the fungal wheat pathogen *Phaeosphaeria nodorum*. Mol Ecol. 2006:15(10):2895–2904. 10.1111/j.1365-294X.2006.02986.x. 16911209

[evaf053-B54] Stukenbrock EH, Mcdonald BA. The origins of plant pathogens in agro-ecosystems. Annu Rev Phytopathol. 2008:46(1):75–100. 10.1146/annurev.phyto.010708.154114. 18680424

[evaf053-B55] Taliadoros D, Feurtey A, Wyatt N, Barrès B, Gladieux P, Friesen TL, Stukenbrock EH. Emergence and spread of the barley net blotch pathogen coincided with crop domestication and cultivation history. PLoS Genet. 2024:20(1):e1010884. 10.1371/journal.pgen.1010884. 38285729 PMC10852282

[evaf053-B56] Taliadoros D, Stukenbrock EH. The use of evolutionary analyses to predict functionally relevant traits in filamentous plant pathogens. Curr Opin Microbiol. 2023:73:102244. 36889024 10.1016/j.mib.2022.102244

[evaf053-B57] Tehseen MM, Poore RC, Fugate KK, Bolton MD, Ramachandran V, Wyatt NA, Li X, Chu C. Potential of publicly available Beta vulgaris germplasm for sustainable sugarbeet improvement indicated by combining analysis of genetic diversity and historic resistance evaluation. Crop Sci. 2023:63(4):2255–2273. 10.1002/csc2.20978.

[evaf053-B58] Thierry M, Charriat F, Milazzo J, Adreit H, Ravel S, Cros-Arteil S, Borron S, Sella V, Kroj T, Ioos R, et al Maintenance of divergent lineages of the Rice Blast Fungus Pyricularia oryzae through niche separation, loss of sex and post-mating geneticin compatibilities. PLoS Pathog. 2022:18(7):e1010687. 35877779 10.1371/journal.ppat.1010687 PMC9352207

[evaf053-B59] Thines M . An evolutionary framework for host shifts—jumping ships for survival. New Phytol. 2019:224(2):605–617. 10.1111/nph.16092. 31381166

[evaf053-B60] Toruño TY, Stergiopoulos I, Coaker G. Plant-pathogen effectors: cellular probes interfering with plant defenses in spatial and temporal manners. Annu Rev Phytopathol. 2016:54(1):419–441. 10.1146/annurev-phyto-080615-100204. 27359369 PMC5283857

[evaf053-B61] Vaghefi N, Kikkert JR, Bolton MD, Hanson LE, Secor GA, Nelson SC, Pethybridge SJ. Global genotype flow in *Cercospora beticola* populations confirmed through genotyping-by-sequencing. PLoS One. 2017:12(10):e0186488. 10.1371/journal.pone.0186488. 29065114 PMC5655429

[evaf053-B62] Vaghefi N, Nelson SC, Kikkert JR, Pethybridge SJ. Genetic structure of *Cercospora beticola* populations on Beta vulgaris in New York and Hawaii. Sci Rep. 2017:7(1):1726. 10.1038/s41598-017-01929-4. 28496148 PMC5431814

[evaf053-B63] Wickham H . ggplot2: elegant graphics for data analysis. New York (NY): Springer-Verlag; 2016.

[evaf053-B64] Zhang C, Dong SS, Xu JY, He WM, Yang TL. PopLDdecay: a fast and effective tool for linkage disequilibrium decay analysis based on variant call format files. Bioinformatics. 2019:35(10):1786–1788. 10.1093/bioinformatics/bty875. 30321304

[evaf053-B65] Zhang J, Kobert K, Flouri T, Stamatakis A. PEAR: a fast and accurate illumina paired-End reAd mergeR. Bioinformatics. 2014:30(5):614–620. 10.1093/bioinformatics/btt593. 24142950 PMC3933873

[evaf053-B66] Zhong Z, Chen M, Lin L, Han Y, Bao J, Tang W, Lin L, Lin Y, Somai R, Lu L, et al Population genomic analysis of the rice blast fungus reveals specific events associated with expansion of three main clades. ISME J. 2018:12(8):1867–1878. 10.1038/s41396-018-0100-6. 29568114 PMC6051997

