# Supplementary material for: Genome-wide Evidence of Host Specialization in Wild and Farmland Populations of the Fungal Leaf Spot Pathogen, Cercospora beticola
Source: Genome Biol Evol. 2025 Apr 28;17(4):evaf053. doi: 10.1093/gbe/evaf053 (PMC12034460; doi:10.1093/gbe/evaf053)
Supplement: evaf053_Supplementary_Data [file evaf053_supplementary_data.pdf]

Genome-wide evidence of host specialization in wild and farmland populations of the Cercospora leaf spot pathogen, Cercospora beticola

Demetris Taliadoros, Lizel Potgieter, Amar Dhiman, Nathan A. Wyatt, Mark McMullan, Christian Jung, Melvin D. Bolton, and Eva Stukenbrock

Supplementary Materials

Table S1: Geographic and host origin, raw read count, percentage of mapped reads and mean coverage per isolate across the genome.

	Country	Region	Host	No of raw reads	% of reads mapped	Mean depth (X)
CRO_MB1.10	Croatia	Antenal	Sea beet	12741624	92,6	21
CRO_MB1.11	Croatia	Antenal	Sea beet	17752382	86,1	30
CRO_MB1.14	Croatia	Antenal	Sea beet	11442291	97,4	28
CRO_MB1.18	Croatia	Antenal	Sea beet	11000849	80,3	31
CRO_MB1.23	Croatia	Antenal	Sea beet	11568080	89,6	17
CRO_MB1.4	Croatia	Antenal	Sea beet	11770110	79,7	19
CRO_MB1.6	Croatia	Antenal	Sea beet	16532286	83,5	21
CRO_MB1.8	Croatia	Antenal	Sea beet	9526989	95,1	14
CRO_MB1.9	Croatia	Antenal	Sea beet	10564533	93,7	20
CRO_MB2.25	Croatia	Jadransko	Sea beet	17317846	82,8	18
CRO_MB2.27	Croatia	Jadransko	Sea beet	15625406	81,9	18
CRO_MB2.28	Croatia	Jadransko	Sea beet	17562211	82,3	14
CRO_MB2.30	Croatia	Jadransko	Sea beet	12207034	86,2	11
CRO_MB2.34	Croatia	Jadransko	Sea beet	10915413	79,9	12
CRO_MB2.36	Croatia	Jadransko	Sea beet	15371702	87,5	28
CRO_MB2.37	Croatia	Jadransko	Sea beet	13619885	96,5	20
CRO_MB2.39	Croatia	Jadransko	Sea beet	13620018	82,5	11
CRO_MB2.40	Croatia	Jadransko	Sea beet	15801129	99,7	16
CRO_MB2.42	Croatia	Jadransko	Sea beet	12748104	93,9	22
CRO_MB2.44	Croatia	Jadransko	Sea beet	11985340	82,4	30
CRO_MB2.47	Croatia	Jadransko	Sea beet	11082026	94	25
CRO_MB2.55	Croatia	Jadransko	Sea beet	17766202	89,8	27
CRO_MB2.57	Croatia	Jadransko	Sea beet	13219281	80,8	29
CRO_MB2.62	Croatia	Jadransko	Sea beet	10020086	90,5	27
DE12-103	Germany	Germany	Sugar beet	12218567	86,1	22
DE12-120	Germany	Germany	Sugar beet	14958668	83,6	28
DE12-123	Germany	Germany	Sugar beet	16530411	88,5	20
DE12-124	Germany	Germany	Sugar beet	16463913	98,1	28
DE12-215	Germany	Germany	Sugar beet	11600345	79,5	31
DE12-58	Germany	Germany	Sugar beet	11853970	86,4	12
DE12-91	Germany	Germany	Sugar beet	14129323	81,5	18
DE_11_58	Germany	Germany	Sugar beet	16436996	79,9	31

DE_11_91	Germany	Germany	Sugar beet	15111571	83,9	23
DK12-232	Denmark	Denmark	Sugar beet	13420946	98,8	21
DK12-290	Denmark	Denmark	Sugar beet	11974634	89,4	15
DK12-304	Denmark	Denmark	Sugar beet	14607639	91,4	19
DK12-305	Denmark	Denmark	Sugar beet	15245069	87,1	13
DK12-307	Denmark	Denmark	Sugar beet	12056615	79,7	17
DK12-310	Denmark	Denmark	Sugar beet	15429294	97,4	26
DK12-316	Denmark	Denmark	Sugar beet	11649969	80,1	11
DK12-317	Denmark	Denmark	Sugar beet	17806925	91,9	11
DK12-319	Denmark	Denmark	Sugar beet	13259274	85,6	12
DK12-322	Denmark	Denmark	Sugar beet	14726383	83,7	20
DK12-325	Denmark	Denmark	Sugar beet	18184676	90,7	17
DK12-327	Denmark	Denmark	Sugar beet	17043332	85,8	23
DK12-338	Denmark	Denmark	Sugar beet	17438574	94,5	15
FR10-1-18	France	France	Sugar beet	17512554	93,6	21
FR10-1-58	France	France	Sugar beet	14224498	89,9	24
FR10-2-65	France	France	Sugar beet	14197760	99,8	18
FR10-2-66	France	France	Sugar beet	17534556	97,9	31
FR10-2-84	France	France	Sugar beet	17364866	86,8	15
FR10-2-90	France	France	Sugar beet	13449740	86,3	31
FR10-3-5	France	France	Sugar beet	12576276	79,5	11
FR10-4-10	France	France	Sugar beet	17536408	81,8	16
FR10-4-23	France	France	Sugar beet	14364728	81,3	25
FR10-4-24	France	France	Sugar beet	9403478	96,9	11
FR10-4-26	France	France	Sugar beet	12030316	89,9	32
GER_135E	Germany	Germany	Sugar beet	13879618	88,5	28
GER_138C	Germany	Germany	Sugar beet	14447678	93,2	25
GER_22E	Germany	Germany	Sugar beet	10198522	89,8	14
GER_71D	Germany	Germany	Sugar beet	9645374	96,1	22
GER_71E	Germany	Germany	Sugar beet	12602786	90,8	27
IT10-10-91	Italy	Italy	Sugar beet	11209152	99,4	11
IT10-14-32	Italy	Italy	Sugar beet	11935052	85,4	26
IT10-6-61	Italy	Italy	Sugar beet	18893832	85,7	29

IT10-7-77	Italy	Italy	Sugar beet	13030634	99,3	27
IT10-8-16	Italy	Italy	Sugar beet	14762336	95,8	26
IT_5.36	Italy	Venezia	Sugar beet	16195280	98,7	18
IT_5.44	Italy	Rovigo	Sugar beet	8253280	98,4	25
IT_5.46	Italy	Rovigo	Sugar beet	8253280	92,4	26
IT_5.52	Italy	Rovigo	Sugar beet	8253280	96	26
IT_5.60	Italy	Rovigo	Sugar beet	8253280	82,3	24
IT_5.69	Italy	Rovigo	Sugar beet	8253280	95,1	17
IT_5.75	Italy	Bologna	Sugar beet	8253280	91	26
IT_5.81	Italy	Bologna	Sugar beet	8253280	85,9	26
IT_5.89	Italy	Bologna	Sugar beet	8253280	83,5	31
IT_5.94	Italy	Ferrara	Sugar beet	8253280	83,8	32
IT_6.14	Italy	Venezia	Sugar beet	8253280	97,7	17
IT_6.28	Italy	Venezia	Sugar beet	9061450	99,4	27
IT_6.4	Italy	Venezia	Sugar beet	8816342	87,3	19
IT_6.51	Italy	Ferrara	Sugar beet	7250004	83,8	12
IT_6.84	Italy	Bologna	Sugar beet	7731792	98,5	14
IT_6.94	Italy	Bologna	Sugar beet	9282346	80,1	27
INDIV_11	USA	Fargo	Sugar beet	9959226	97,8	30
INDIV_12	USA	Fargo	Sugar beet	9447140	94,7	18
INDIV_13	USA	Fargo	Sugar beet	8063272	94,3	25
INDIV_14	USA	Fargo	Sugar beet	8341094	84,9	16
INDIV_15	USA	Fargo	Sugar beet	7602410	89,4	14
INDIV_16	USA	Fargo	Sugar beet	8317210	84,9	11
INDIV_17	USA	Fargo	Sugar beet	10074954	84,2	22
INDIV_18	USA	Fargo	Sugar beet	8453636	97,9	32
INDIV_19	USA	Fargo	Sugar beet	8706804	99,9	27
INDIV_1	USA	Fargo	Sugar beet	10006100	93,4	29
INDIV_20	USA	Fargo	Sugar beet	9631512	88,8	29
INDIV_21	USA	Fargo	Sugar beet	17126014	80,2	26
INDIV_22	USA	Fargo	Sugar beet	14155222	92,8	11
INDIV_23	USA	Fargo	Sugar beet	14810636	87,2	28
INDIV_24	USA	Fargo	Sugar beet	11770764	98,5	12

INDIV_25	USA	Fargo	Sugar beet	14054934	86,2	23
INDIV_26	USA	Fargo	Sugar beet	10433228	92,1	22
INDIV_27	USA	Fargo	Sugar beet	13006502	90	11
INDIV_28	USA	Fargo	Sugar beet	13214536	96,1	21
INDIV_29	USA	Fargo	Sugar beet	14272216	84,9	11
INDIV_2	USA	Fargo	Sugar beet	12381302	79,6	32
INDIV_30	USA	Fargo	Sugar beet	12314736	89,4	11
INDIV_31	USA	Fargo	Sugar beet	12510086	95,7	16
INDIV_32	USA	Fargo	Sugar beet	9682212	92,2	15
INDIV_34	USA	Fargo	Sugar beet	12350766	95,6	13
INDIV_35	USA	Fargo	Sugar beet	10866418	84,2	15
INDIV_36	USA	Fargo	Sugar beet	15822832	98,9	19
INDIV_38	USA	Fargo	Sugar beet	11925270	82,7	30
INDIV_39	USA	Fargo	Sugar beet	10900204	81	11
INDIV_3	USA	Fargo	Sugar beet	13069202	92,7	31
INDIV_40	USA	Fargo	Sugar beet	12201556	97	18
INDIV_41	USA	Fargo	Sugar beet	12674092	87,6	20
INDIV_42	USA	Fargo	Sugar beet	13832062	99,7	20
INDIV_44	USA	Fargo	Sugar beet	11367388	81,4	15
INDIV_45	USA	Fargo	Sugar beet	9782152	86,9	14
INDIV_46	USA	Fargo	Sugar beet	12962802	87,2	15
INDIV_48	USA	Fargo	Sugar beet	13631672	79,2	26
INDIV_49	USA	Fargo	Sugar beet	14638470	94,4	23
INDIV_4	USA	Fargo	Sugar beet	14638470	82,2	31
INDIV_50	USA	Fargo	Sugar beet	15387752	93,5	26
INDIV_54	USA	Fargo	Sugar beet	14976766	88,4	15
INDIV_55	USA	Fargo	Sugar beet	11998192	95,3	31
INDIV_57	USA	Fargo	Sugar beet	13430048	85,9	29
INDIV_58	USA	Fargo	Sugar beet	12550804	82,4	26
INDIV_59	USA	Fargo	Sugar beet	13951680	95,7	11
INDIV_5	USA	Fargo	Sugar beet	11811852	99	22
INDIV_62	USA	Fargo	Sugar beet	11877672	92,8	25
INDIV_63	USA	Fargo	Sugar beet	13273322	85,9	12

INDIV_65	USA	Fargo	Sugar beet	14795006	98,4	21
INDIV_66	USA	Fargo	Sugar beet	13838612	88,1	28
INDIV_67	USA	Fargo	Sugar beet	11976570	96,8	19
INDIV_68	USA	Fargo	Sugar beet	13824020	94,6	30
INDIV_69	USA	Fargo	Sugar beet	12253114	96,2	22
INDIV_6	USA	Fargo	Sugar beet	10925746	93,8	22
INDIV_71	USA	Fargo	Sugar beet	12693346	85,5	31
INDIV_72	USA	Fargo	Sugar beet	13426800	99,6	21
INDIV_73	USA	Fargo	Sugar beet	13226236	96,4	14
INDIV_74	USA	Fargo	Sugar beet	12082990	95,7	20
INDIV_75	USA	Fargo	Sugar beet	13754388	91,4	29
INDIV_76	USA	Fargo	Sugar beet	11176670	90,1	25
INDIV_77	USA	Fargo	Sugar beet	10608698	79,5	12
INDIV_78	USA	Fargo	Sugar beet	13999678	91,6	15
INDIV_7	USA	Fargo	Sugar beet	14557434	94,7	13
INDIV_80	USA	Fargo	Sugar beet	12252820	79,6	28
INDIV_81	USA	Fargo	Sugar beet	12465424	98	27
INDIV_82	USA	Fargo	Sugar beet	12468574	85,4	30
INDIV_83	USA	Fargo	Sugar beet	16962372	94	17
INDIV_84	USA	Fargo	Sugar beet	16984706	91,1	29
INDIV_85	USA	Fargo	Sugar beet	16951766	81,8	14
INDIV_86	USA	Fargo	Sugar beet	16945342	92,4	12
INDIV_87	USA	Fargo	Sugar beet	16690702	92,2	18
INDIV_88	USA	Fargo	Sugar beet	16920682	84,4	26
INDIV_89	USA	Fargo	Sugar beet	14550292	92	20
INDIV_90	USA	Fargo	Sugar beet	13437872	81,9	21
INDIV_91	USA	Fargo	Sugar beet	11394620	80	12
INDIV_92	USA	Fargo	Sugar beet	13359108	80,9	25
INDIV_93	USA	Fargo	Sugar beet	13048092	85,6	28
INDIV_94	USA	Fargo	Sugar beet	11409618	80,8	11
INDIV_95	USA	Fargo	Sugar beet	11021390	92,5	29
INDIV_9	USA	Fargo	Sugar beet	13223262	79,1	26
NL12-105	Netherlands	Netherlands	Sugar beet	9963172	95,3	16

NL12-340	Netherlands	Netherlands	Sugar beet	12016942	87,7	22
NL12-341	Netherlands	Netherlands	Sugar beet	10776176	79,5	23
NL12-343	Netherlands	Netherlands	Sugar beet	17076188	82,8	19
NL12-344	Netherlands	Netherlands	Sugar beet	17063134	92,8	27
NL12-350	Netherlands	Netherlands	Sugar beet	17075834	89,9	14
NL12-352	Netherlands	Netherlands	Sugar beet	17071394	92,6	20
NL12-354	Netherlands	Netherlands	Sugar beet	17083040	88,7	29
NL12-357	Netherlands	Netherlands	Sugar beet	17075942	91	11
NL12-361	Netherlands	Netherlands	Sugar beet	17075076	96,2	25
NL12-366	Netherlands	Netherlands	Sugar beet	17059976	99,7	25
NL12-372	Netherlands	Netherlands	Sugar beet	17042128	93,4	29
NL12-381	Netherlands	Netherlands	Sugar beet	17055246	84,7	23
NL12-392	Netherlands	Netherlands	Sugar beet	17060092	79,6	24
NL12-418	Netherlands	Netherlands	Sugar beet	17049136	89,7	11
NY_545	USA	New York	Table beet	17075416	94,3	21
NY_546	USA	New York	Table beet	17075752	88,8	13
NY_547	USA	New York	Table beet	17081536	90,6	14
NY_557	USA	New York	Table beet	17077874	92,3	13
NY_558	USA	New York	Table beet	17076540	96,3	15
NY_559	USA	New York	Table beet	17080464	87	13
NY_560	USA	New York	Table beet	7982178	97,1	28
NY_561	USA	New York	Table beet	8004992	86	13
NY_562	USA	New York	Table beet	9427296	82,6	32
NY_563	USA	New York	Table beet	8002446	85,1	28
NY_564	USA	New York	Table beet	8003704	87,2	22
NY_574	USA	New York	Table beet	8012654	86,6	15
NY_575	USA	New York	Table beet	9457908	90,6	15
NY_576	USA	New York	Table beet	9591510	87,8	15
NY_581	USA	New York	Table beet	8066482	87,8	15
NY_584	USA	New York	Table beet	8042390	96,2	19
NY_585	USA	New York	Table beet	8010294	93,9	29
NY_587	USA	New York	Table beet	7994650	87,9	15
NY_588	USA	New York	Table beet	9525006	90,5	25

NY_589	USA	New York	Table beet	9525072	90,3	32
NY_590	USA	New York	Table beet	9560652	81,9	16
NY_591	USA	New York	Table beet	7991714	89,3	32
NY_592	USA	New York	Table beet	8029974	95,5	28
NY_593	USA	New York	Table beet	9376518	99,8	28
SCL15-1-11	Chile	Chile	Fodder beet	9294962	89,9	30
SCL15-2_95	Chile	Chile	Fodder beet	9434238	94,9	29
SCL15-3-26	Chile	Chile	Fodder beet	9264502	91,9	29
SCL15-3-52	Chile	Chile	Fodder beet	9253524	87,1	25
SCL15-3-85	Chile	Chile	Fodder beet	9323786	97,7	22
SCL15-3-87	Chile	Chile	Fodder beet	9261130	85,8	23
SCL15-4-10	Chile	Chile	Fodder beet	9254410	87,4	26
SCL15-4_11	Chile	Chile	Fodder beet	9315246	90,8	12
SE12-144	Sweden	Sweden	Sugar beet	9078076	99,3	23
SE12-261	Sweden	Sweden	Sugar beet	9175852	82,5	24
SE12-264	Sweden	Sweden	Sugar beet	9255352	83,4	29
SE12-269	Sweden	Sweden	Sugar beet	9261186	86,7	14
SE_11_269	Sweden	Sweden	Sugar beet	9381454	95,7	26
SE_12-174	Sweden	Sweden	Sugar beet	9300908	95,7	26
SE_12_141	Sweden	Sweden	Sugar beet	9278624	88,8	29
SE_12_153	Sweden	Sweden	Sugar beet	9375614	83,4	28
SE_12_166	Sweden	Sweden	Sugar beet	9268318	84,1	12
SE_12_180	Sweden	Sweden	Sugar beet	9294476	98,8	18
SE_12_188	Sweden	Sweden	Sugar beet	9381910	80,9	31
SE_12_87	Sweden	Sweden	Sugar beet	9065884	91,3	24
SP12-100	Spain	Spain	Sugar beet	9369738	93,3	28
SP12-107	Spain	Spain	Sugar beet	9293850	92,3	29
SP12-111	Spain	Spain	Sugar beet	9341446	82	22
SP12-120	Spain	Spain	Sugar beet	12116483	83,6	15
SP12-15	Spain	Spain	Sugar beet	8606422	94,8	19
SP12-16	Spain	Spain	Sugar beet	17052705	79,4	22
SP12-3	Spain	Spain	Sugar beet	10641498	99,2	29
SP12-35	Spain	Spain	Sugar beet	11455354	97,5	27

SP12-41	Spain	Spain	Sugar beet	15756417	82,2	19
SP12-46	Spain	Spain	Sugar beet	17364843	92,4	19
SP12-52	Spain	Spain	Sugar beet	9746955	95	19
SP12-6	Spain	Spain	Sugar beet	9888387	95,6	14
SP12-61	Spain	Spain	Sugar beet	16697113	96,9	14
SP12-65	Spain	Spain	Sugar beet	16477998	99,4	19
SP12-68	Spain	Spain	Sugar beet	11911981	83,4	19
SP12-69	Spain	Spain	Sugar beet	15552906	99,8	32
SP12-79	Spain	Spain	Sugar beet	15383784	98,1	14
SP12-90	Spain	Spain	Sugar beet	15880404	93,4	25
SP12-91	Spain	Spain	Sugar beet	10771012	84,4	20
SP12-96	Spain	Spain	Sugar beet	12495635	99,8	24
TK12-103	Turkey	Turkey	Sugar beet	12131859	82,6	19
TK12-372	Turkey	Turkey	Sugar beet	17589587	82,2	23
TK12-373	Turkey	Turkey	Sugar beet	11302657	81,7	18
TK12-380	Turkey	Turkey	Sugar beet	10124962	95,6	28
TK12-384	Turkey	Turkey	Sugar beet	15177314	88,6	30
TK12-385	Turkey	Turkey	Sugar beet	13446874	96,5	14
TK12-438	Turkey	Turkey	Sugar beet	10833605	96,1	13
TK12-468	Turkey	Turkey	Sugar beet	17435536	98,4	32
TK12-47	Turkey	Turkey	Sugar beet	17321293	85,3	28
TK12-470	Turkey	Turkey	Sugar beet	10578209	96,1	22
TK12-471	Turkey	Turkey	Sugar beet	12748355	84,6	20
TK12-473	Turkey	Turkey	Sugar beet	10661161	85,1	30
TK12-581	Turkey	Turkey	Sugar beet	11553648	81,9	22
TK12-79	Turkey	Turkey	Sugar beet	13383999	91,4	25
TK12-80	Turkey	Turkey	Sugar beet	11814810	83,5	28
TK12-83	Turkey	Turkey	Sugar beet	9626762	95	23
TK12-84	Turkey	Turkey	Sugar beet	11817686	81,7	14
TK12-85	Turkey	Turkey	Sugar beet	15430934	92	11
UK11-24	UK	UK	Sugar beet	12287438	84	14
UK11-79	UK	UK	Sugar beet	93249893	85,4	16
UK_10.2.2B	UK	Southwold	Sea beet	8389284	89,7	19

UK_1A5.2B	UK	Southwold	Sea beet	11076620	89,8	29
UK_21.5.1A	UK	Ordorf	Sea beet	2097850	86,7	18
UK_21.5.1B	UK	Ordorf	Sea beet	2097850	99,7	16
UK_21.8.1A	UK	Ordorf	Sea beet	6588928	98,3	26
UK_23.2.1B	UK	Ordorf	Sea beet	18180142	88,1	20
UK_23.6.2A	UK	Ordorf	Sea beet	14570712	86,4	30
UK_24.2.1A	UK	Ordorf	Sea beet	13944818	91,6	12
UK_24.4.1B	UK	Ordorf	Sea beet	9928558	98	26
UK_25.1.1A	UK	Ordorf	Sea beet	7840580	84	13
UK_25.1.2A	UK	Ordorf	Sea beet	17680908	99,3	26
UK_26.8.1	UK	Ordorf	Sea beet	13725036	89,8	27
UK_27.7.2B	UK	Ordorf	Sea beet	13559130	96,5	14
UK_29.5.1B	UK	Ordorf	Sea beet	9344572	84,5	31
UK_2B10.1A	UK	Southwold	Sea beet	12055598	99,8	18
UK_2B10.1B	UK	Southwold	Sea beet	13307111	95,1	20
UK_3.5.1	UK	Southwold	Sea beet	8641994	96,8	23
UK_30.5.2B	UK	Ordorf	Sea beet	11831242	87,1	14
UK_30.7.2B	UK	Ordorf	Sea beet	12884536	85,4	27
UK_4A2.1B	UK	Southwold	Sea beet	14212344	80,5	14
UK_4A3.1B	UK	Southwold	Sea beet	10469720	91,9	31
UK_4A3.2A	UK	Southwold	Sea beet	9836742	84,8	26
UK_4A8.1B	UK	Southwold	Sea beet	12652340	96,7	30
UK_4A8.2B	UK	Southwold	Sea beet	9619922	94,3	24
UK_4A9.1A	UK	Southwold	Sea beet	8496690	92,8	32
UK_4B5.1B	UK	Southwold	Sea beet	10081402	84,6	16
UK_4B9.2A	UK	Southwold	Sea beet	13290370	79,2	23
UK_5.7.2A	UK	Southwold	Sea beet	9662206	88,5	31
UK_51.7.1	UK	Bawdsey	Sea beet	17632584	94,5	13
UK_53A7.2	UK	Bawdsey	Sea beet	1816710	86	21
UK_54.6.1B	UK	Bawdsey	Sea beet	12153990	81,5	15
UK_55A11.1	UK	Bawdsey	Sea beet	8545020	86,8	21
UK_55A3A	UK	Bawdsey	Sea beet	13288588	86,4	17
UK_55A9A	UK	Bawdsey	Sea beet	10080938	81,3	27
UK_56.12.1B_RS1	UK	Bawdsey	Sea beet	10586376	99,6	32
UK_56.2.2	UK	Bawdsey	Sea beet	8510832	86,8	17
UK_57.1.2A	UK	Bawdsey	Sea beet	5826000	81,1	15
UK_57.1.2B	UK	Bawdsey	Sea beet	4269324	97,5	19
UK_8A11.1	UK	Southwold	Sea beet	17470230	90,2	17

UK_8A11.2B	UK	Southwold	Sea beet	13092496	79,8	19
UK_8B.3.2B	UK	Southwold	Sea beet	16932138	93,9	17
UK_9.10.1A	UK	Southwold	Sea beet	14315192	93,3	11
UK_SUGAR_A	UK	Bracebridge	Sugar beet	15765156	98,7	12
UK_SUGAR_B	UK	Bracebridge	Sugar beet	14163535	99,3	19
UK_SUGAR_C	UK	Bracebridge	Sugar beet	15700555	96,1	30
UK_SUGAR_D	UK	Bracebridge	Sugar beet	16839778	87	25
UK_SUGAR_E	UK	Bracebridge	Sugar beet	8664850	82,4	29
UK_SUGAR_F	UK	Bracebridge	Sugar beet	18076342	84	26
UK_SUGAR_G	UK	Bracebridge	Sugar beet	16203497	87,6	18
UK_SUGAR_H	UK	Bracebridge	Sugar beet	13215400	96,2	23
UK_SUGAR_I	UK	Bracebridge	Sugar beet	13858254	90,3	31
UK_SUGAR_J	UK	Bracebridge	Sugar beet	17114799	89	22
UK_SUGAR_K	UK	Bracebridge	Sugar beet	13548032	99,2	23
UK_SUGAR_L	UK	Bracebridge	Sugar beet	11438991	79,3	19
UK_SUGAR_M	UK	Bracebridge	Sugar beet	15357571	92,1	25
UK_SUGAR_O	UK	Bracebridge	Sugar beet	15355221	93,3	28
UK_SUGAR_P	UK	Bracebridge	Sugar beet	15122121	80,8	26
UK_SUGAR_Q	UK	Bracebridge	Sugar beet	11913126	81,4	24
UK_SUGAR_R	UK	Bracebridge	Sugar beet	9141675	88,4	30
UK_SUGAR_S	UK	Bracebridge	Sugar beet	13681952	91,3	25
UK_SUGAR_T	UK	Bracebridge	Sugar beet	14710020	90,1	29
UK_SUGAR_U	UK	Bracebridge	Sugar beet	15030069	88,2	20
UK_SUGAR_V	UK	Bracebridge	Sugar beet	15301528	91,6	23
UK_SUGAR_W	UK	Bracebridge	Sugar beet	13654640	92,8	29

Table S2:Pairwise wilcoxon test of pairwise nuclatide diferences between populations

	Bawd_uk	CHL	Cro	DE	DK	FR	IT	ND	NL	NY	Ordf_UK	SE	SP	Swd_UK	TK	uk_sea
CHL	0,01266483	NA	NA	NA	NA	NA	NA	NA	NA	NA	NA	NA	NA	NA	NA	NA
Cro	2,11E-14	9,56E-31	NA	NA	NA	NA	NA	NA	NA	NA	NA	NA	NA	NA	NA	NA
DE	1	0,0601532	1,23E-16	NA	NA	NA	NA	NA	NA	NA	NA	NA	NA	NA	NA	NA
DK	1,14E-44	1,74E-22	3,42E-109	4,01E-43	NA	NA	NA	NA	NA	NA	NA	NA	NA	NA	NA	NA
FR	1	1	1,47E-18	1	1,54E-36	NA	NA	NA	NA	NA	NA	NA	NA	NA	NA	NA
IT	0,56588181	1	3,10E-27	1	1,56E-29	1	NA	NA	NA	NA	NA	NA	NA	NA	NA	NA
ND	1	1	3,06E-24	1	1,29E-33	1	1	NA	NA	NA	NA	NA	NA	NA	NA	NA
NL	0,09881652	1	8,79E-30	0,3944368	1,81E-27	1	1	1	NA	NA	NA	NA	NA	NA	NA	NA
NY	1	0,01546926	2,81E-15	1	1,98E-45	1	0,60803591	1	0,11034811	NA	NA	NA	NA	NA	NA	NA
Ordf_UK	9,04E-85	3,23E-52	1,04E-163	1,28E-82	1,32E-05	6,71E-73	7,91E-64	2,38E-70	1,73E-61	7,79E-86	NA	NA	NA	NA	NA	NA
SE	1	0,43673162	1,65E-19	1	4,28E-40	1	1	1	1	1	7,37E-79	NA	NA	NA	NA	NA
SP	2,40E-30	3,11E-51	0,00571013	5,36E-34	3,11E-141	9,09E-36	2,09E-47	6,08E-44	7,83E-51	3,73E-32	1,42E-198	5,38E-38	NA	NA	NA	NA
Swd_UK	0,06813206	3,38E-11	0,00023722	0,00879424	2,23E-69	0,00041801	1,80E-08	6,82E-07	5,46E-10	0,03734684	1,03E-117	0,00044718	1,49E-15	NA	NA	NA
TK	1	7,04E-05	3,48E-11	1	2,56E-53	1	0,00829012	0,09100252	0,0008201	1	2,17E-97	1	4,06E-26	1	NA	NA
uk_sea	0,00973638	6,47E-13	0,00196582	0,00090097	4,92E-74	3,53E-05	4,78E-10	2,59E-08	1,15E-11	0,00458604	7,10E-124	3,02E-05	3,55E-14	1	0,41350568	NA
uk_sugar	2,06E-18	3,83E-05	2,35E-67	3,22E-17	7,26E-06	2,91E-13	1,06E-08	9,39E-11	2,36E-07	1,15E-18	2,08E-26	3,49E-15	7,17E-95	9,94E-36	8,03E-24	6,40E-39

Table S3:Pairwise wilcoxon test of Tajima's D diferences between populations

	NY_Tab	CHL	ND	SP	TK	SE	DK	DE	NL	FR	IT	UK_Sugar	UK_Sea	Stw	Bawd	Ord
CHL	2,84E-04	NA	NA	NA	NA	NA	NA	NA	NA	NA	NA	NA	NA	NA	NA	NA
ND	2,14E-05	1	NA	NA	NA	NA	NA	NA	NA	NA	NA	NA	NA	NA	NA	NA
SP	3,08E-117	3,63E-45	4,60E-28	NA	NA	NA	NA	NA	NA	NA	NA	NA	NA	NA	NA	NA
TK	4,46E+06			1,02E-63	NA	NA	NA	NA	NA	NA	NA	NA	NA	NA	NA	NA
SE	8,25E+08			1,07E-61	1	NA	NA	NA	NA	NA	NA	NA	NA	NA	NA	NA
DK	7,35E-143	4,93E-76	1,65E-52	1,49E+08	1,10E-88	1,88E-86	NA	NA	NA	NA	NA	NA	NA	NA	NA	NA
DE	8,79E+09			3,45E-71	1	1	1,54E-95	NA	NA	NA	NA	NA	NA	NA	NA	NA
NL	1,10E+05	1		1,76E-54	1	1	1,40E-76	1	NA	NA	NA	NA	NA	NA	NA	NA
FR	3,17E+03	1		1,83E-62	1	1	2,53E-92	1	1	NA	NA	NA	NA	NA	NA	NA
IT	9,98E-06	1	1	1,04E-38			2,15E-57		1		NA	NA	NA	NA	NA	NA
UK_Sugar	3,36E+02	2,12E-45	1,54E-35	1,99E-164	1,01E-24	2,00E-20	1,41E-186	4,66E-19	3,80E-29	3,10E-32	3,40E-41	NA	NA	NA	NA	NA
UK_Sea	1,73E-26	2,79E-98	1,50E-82	7,78E-256	5,58E-69	2,53E-61	5,71E-275	1,17E-59	6,11E-74	1,91E-79	7,83E-92	2,57E+08	NA	NA	NA	NA
Stw	2,09E+01	1,12E-53	4,76E-47	1,64E-205	1,66E-30	1,20E-23	1,80E-222	1,58E-22	6,97E-34	1,53E-37	3,74E-50	1	2,53E+05	NA	NA	NA
Bawd		4,30E-24	2,05E-23	3,15E-158	1,40E-08	9,72E-04	1,07E-182	1,47E-02	2,87E-11	2,09E-13	1,92E-24	4,69E+08	2,29E-12		NA	NA
Ord	4,54E-81	3,36E-12	4,04E+01	1,61E-05	3,05E-27	3,95E-33	1,13E-21	1,02E-37	9,01E-27	4,90E-28	2,67E-09	2,30E-133	7,20E-232	8,32E-188	3,61E-137	NA
Croatia	4,97E-46	1,03E+03	1,11E+09	5,55E-09	2,41E-09	3,21E-10	5,96E-31	8,68E-15	1,66E-05	7,50E-07	3,53E+05	9,44E-94	4,41E-168	3,18E-120	2,87E-80	1

Table S4: Proportion of eachmating type per population.

POPULATION	Mat1	Mat2	Mat1/Mat2	P-value*	significant
TK	12	6	2	0,65	-
SW	4	8	0,5	0,73	-
DK	8	4	2,25	0,86	-
NL	5	10	0,5	0,39	-
GE	6	8	0,75	0,59	-
FR	5	6	0,83	0,48	-
SP	9	10	0,83	0,57	-
IT	13	8	1,75	0,66	-
CR	13	10	1,4	0,61	-
BB	11	11	1	0,39	-
OR	1	12	0,08	0,001	*
BD	3	7	0,43	0,63	-
SW	10	7	1,57	0,67	-
ND	36	44	0,8	0,58	-
NY	8	16	0,5	0,35	-
CH	3	4	0,6	0,38	-

* Chi squared test, a=0,05

Table S5: Cross-validation error over 10 replicate runs, the average error per K-value and the standard deviation.

VALUE	CV-error
K1	0,74355
K2	0,692
K3	0,65865
K4	0,64359
K5	0,63989
K6	0,63679
K7	0,64621
K8	0,63658
K9	0,63535
K10	0,64009
K11	0,63599
K12	0,6463
K13	0,65068
K14	0,65674
K15	0,65685
K16	0,66247
K17	0,66655
K18	0,70027
K19	0,70097
K20	0,6965

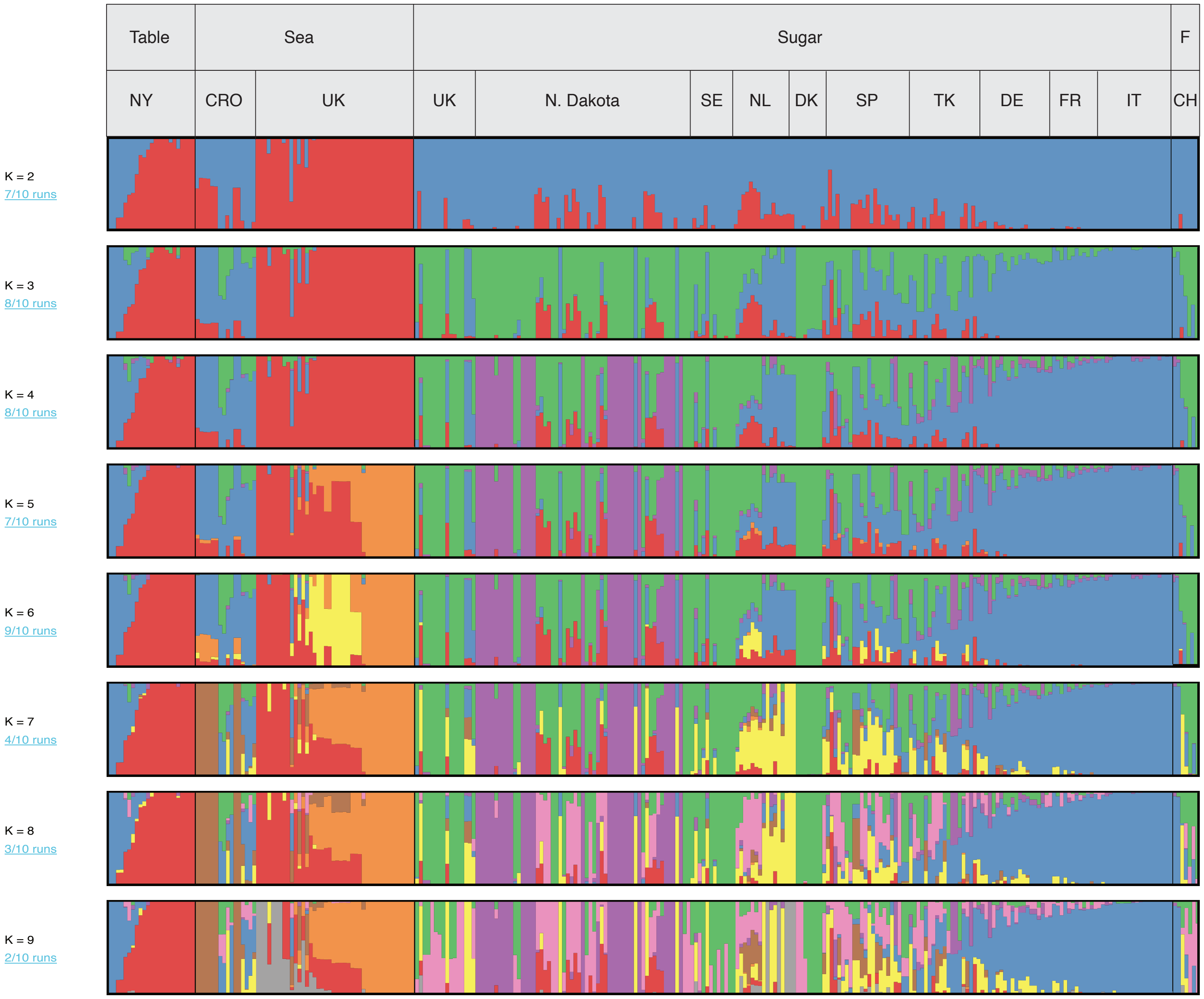

Figure S1: Admixture analysis ran for a range (1-10) of hypothe=cal ancestral groups.

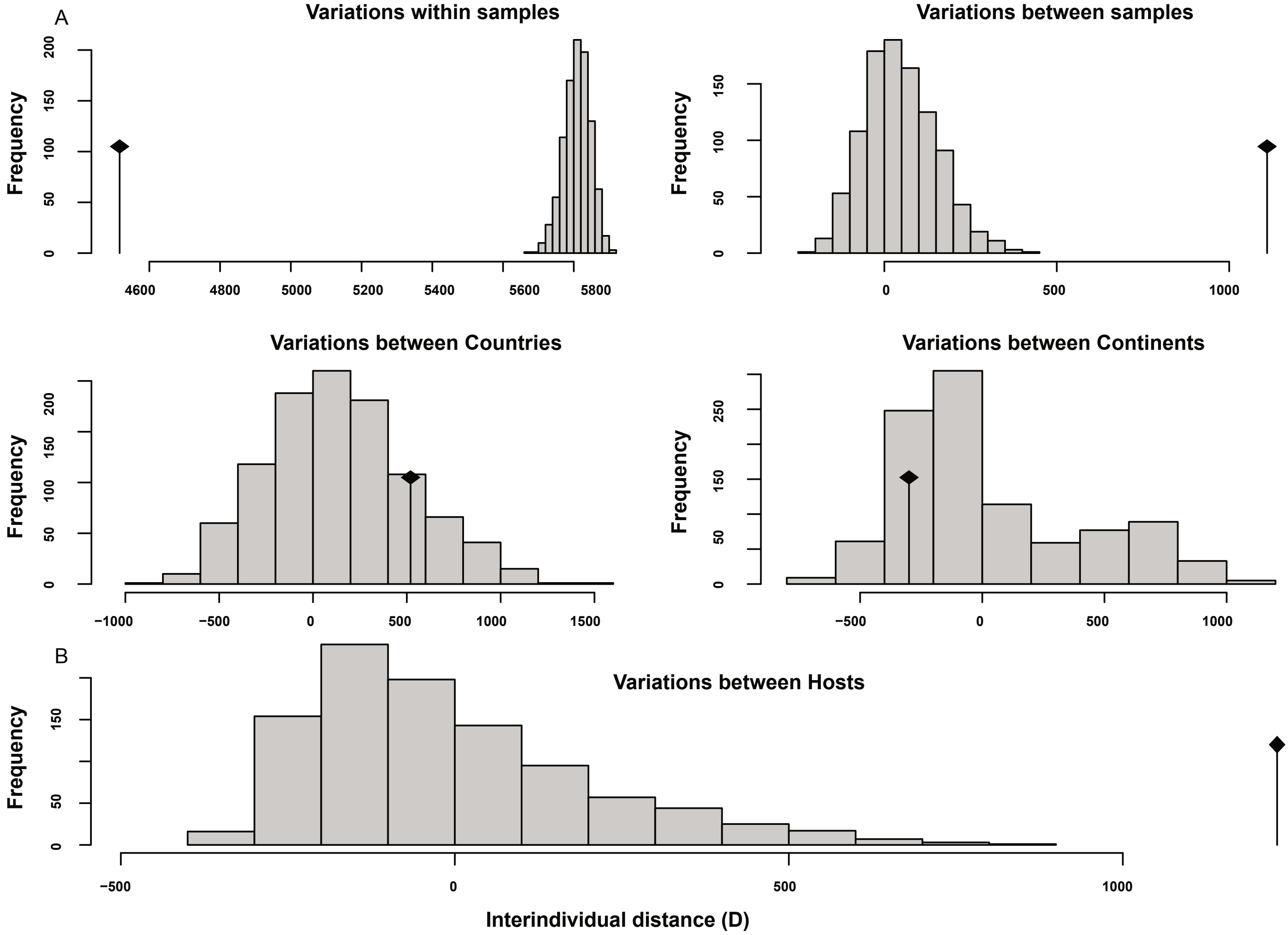

Figure S2: **Analysis of molecular variance within and among *Cercsopora beticola* populations.** Null distribution of the molecular variance components obtained from random permutations of the squared inter-individual distances (D) matrix. The black line with diamonds represents the observed D value. A) Assessment of the significance of country and continent as factors explaining genetic structure. B) Assessment of the significance of the host as a factor explaining genetic structure.

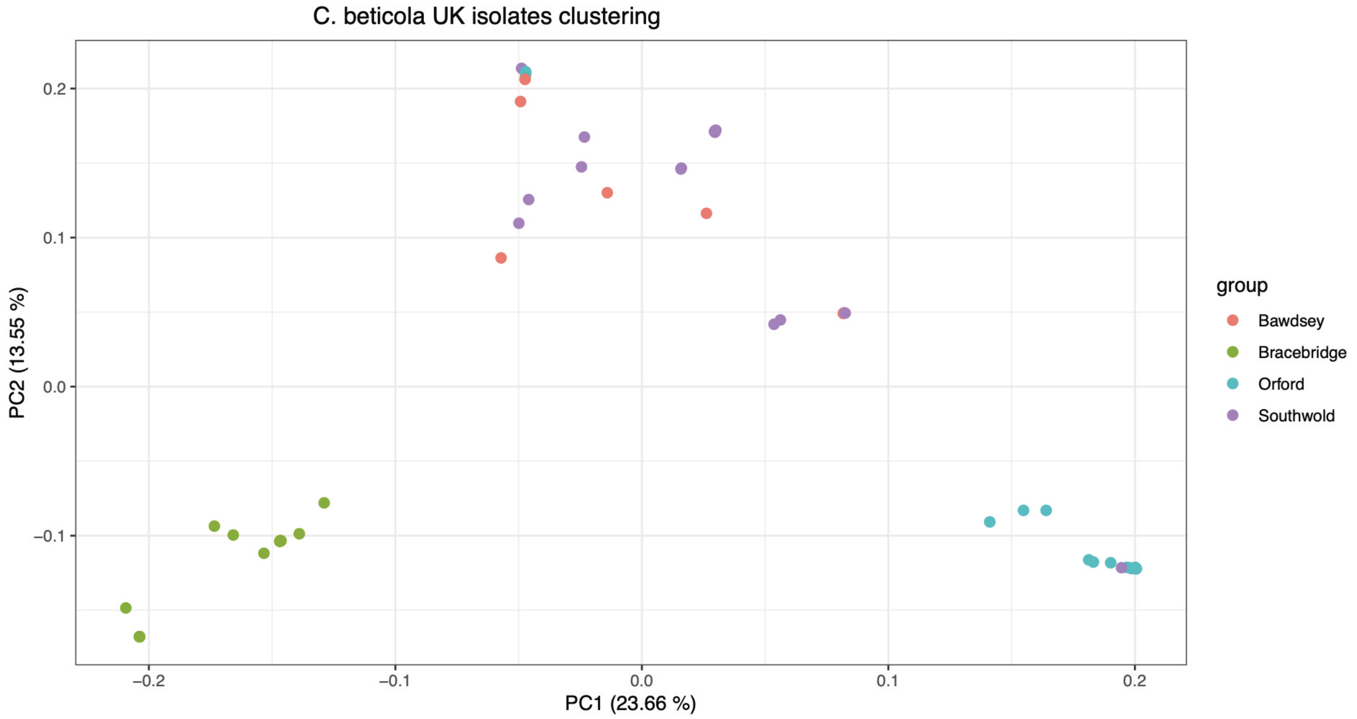

Figure S3: PCA analysis. Color reflects the geographic origin of each isolate was obtained.

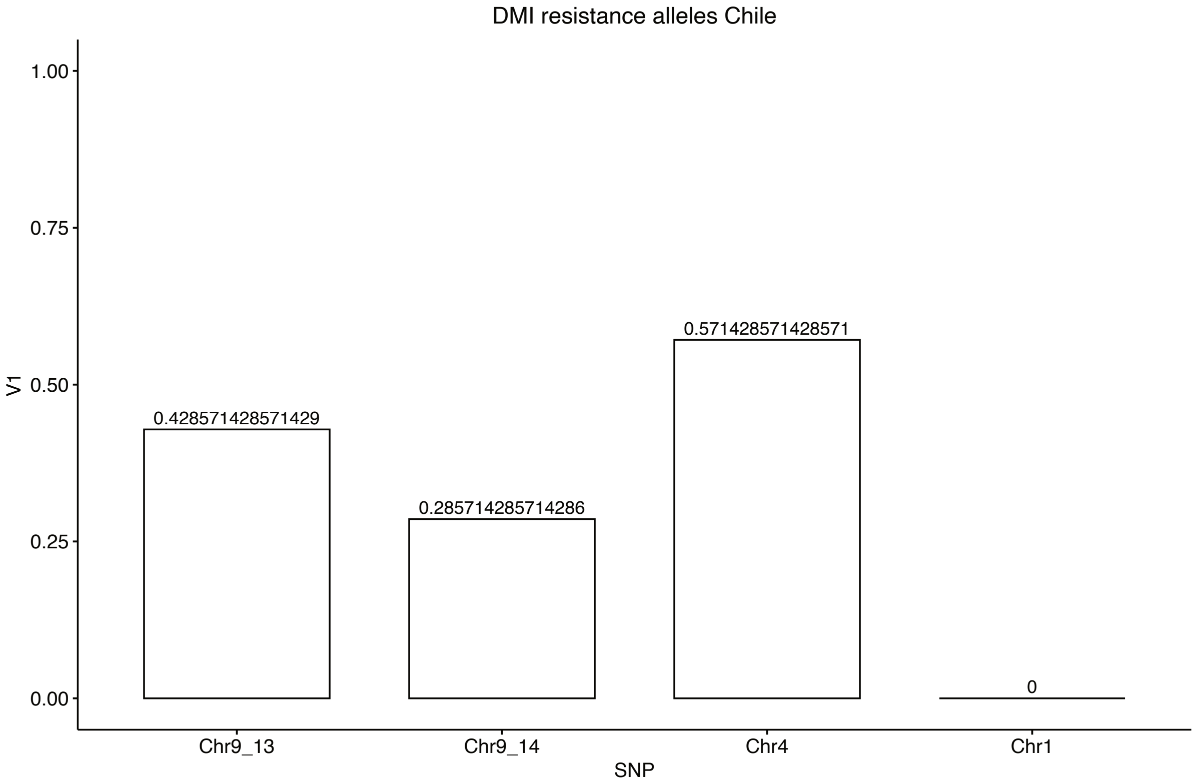

Figure S4: Frequency of DMI resistance associated markers in the Chilian populaton

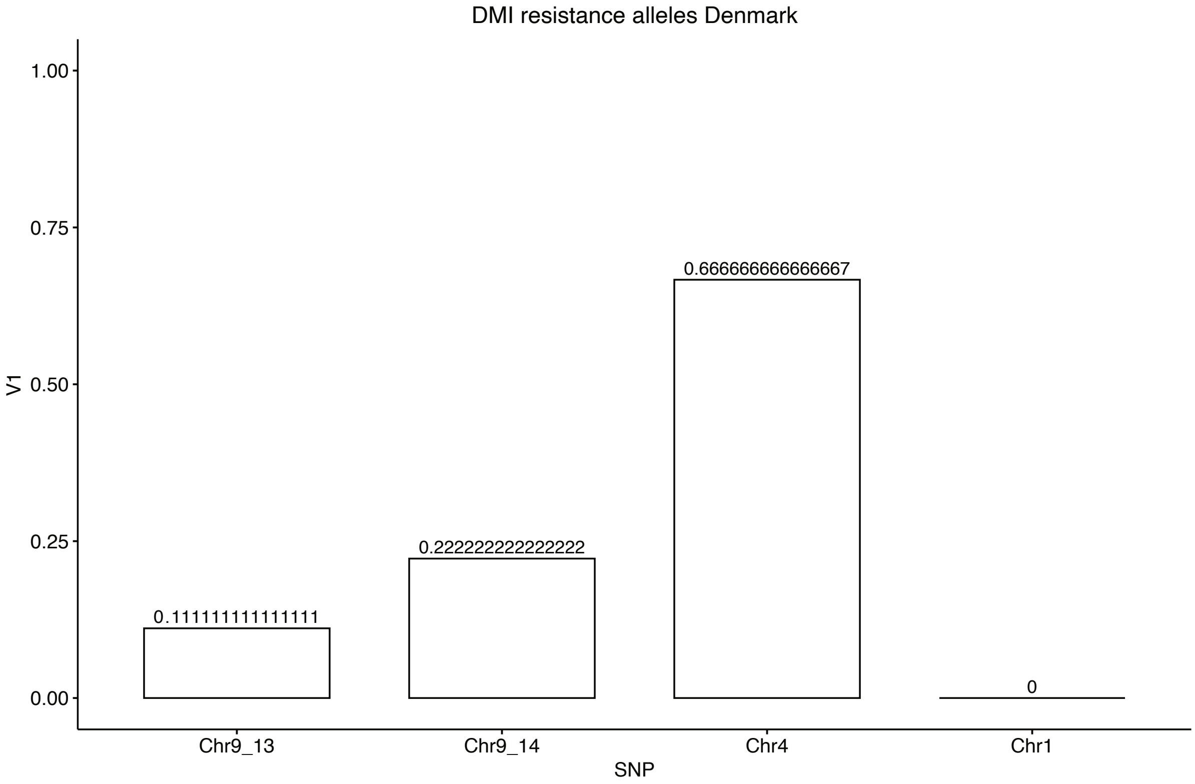

Figure S5: Frequency of DMI resistance associated markers in the Danish populaton

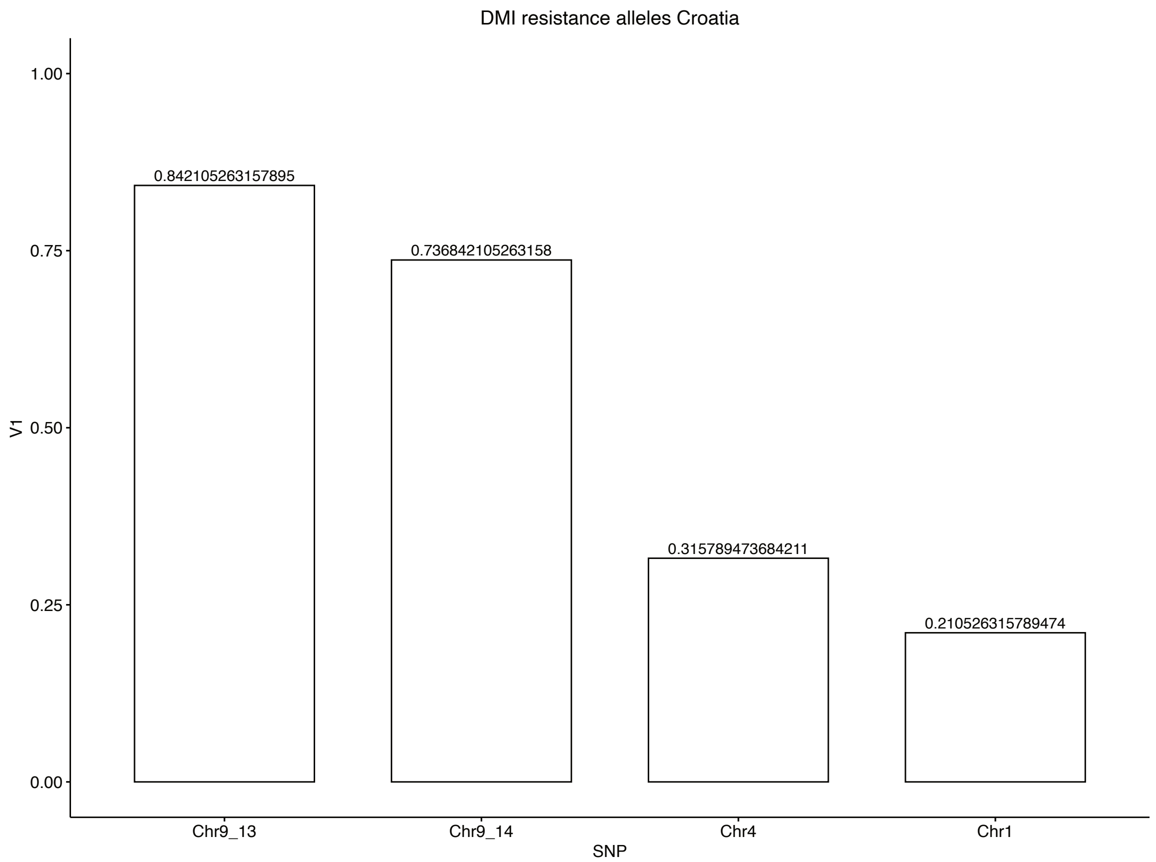

Figure S6: Frequency of DMI resistance associated markers in the Croatian populaton

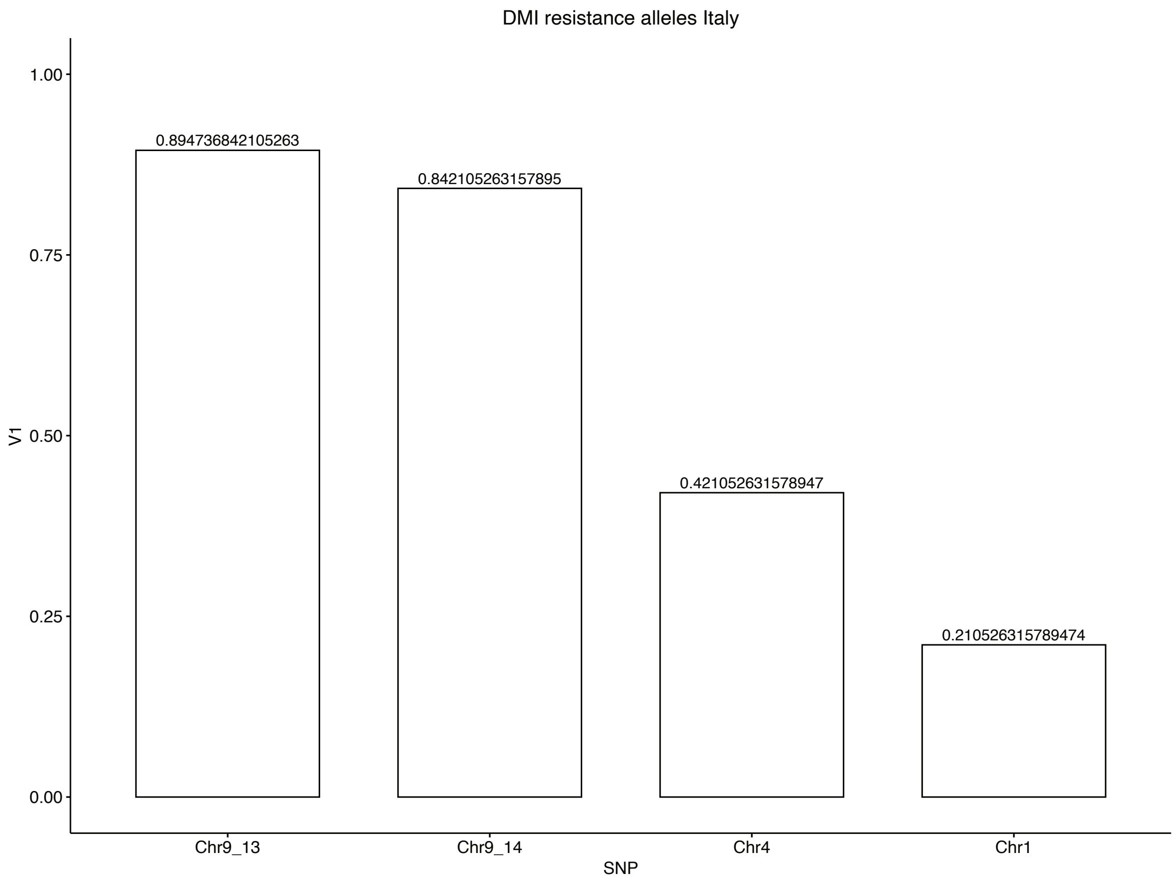

Figure S7: Frequency of DMI resistance associated markers in the Italian populaton

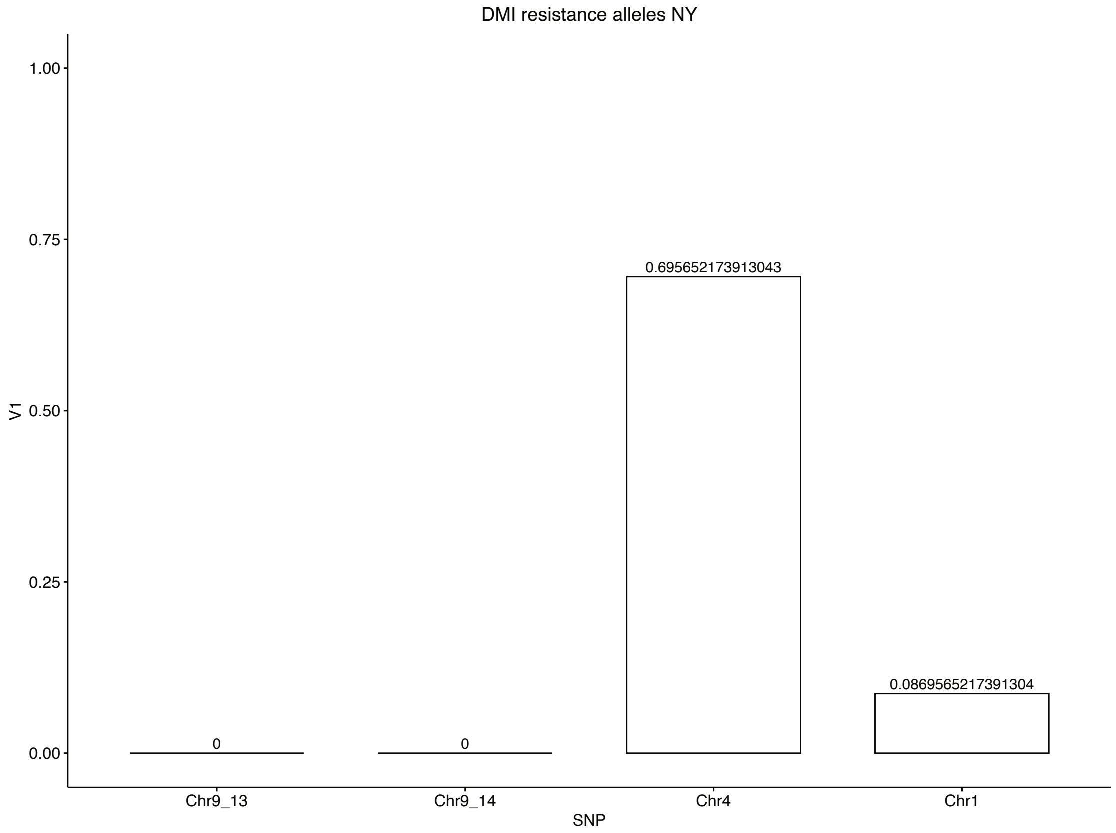

Figure S8: Frequency of DMI resistance associated markers in the New york populaton

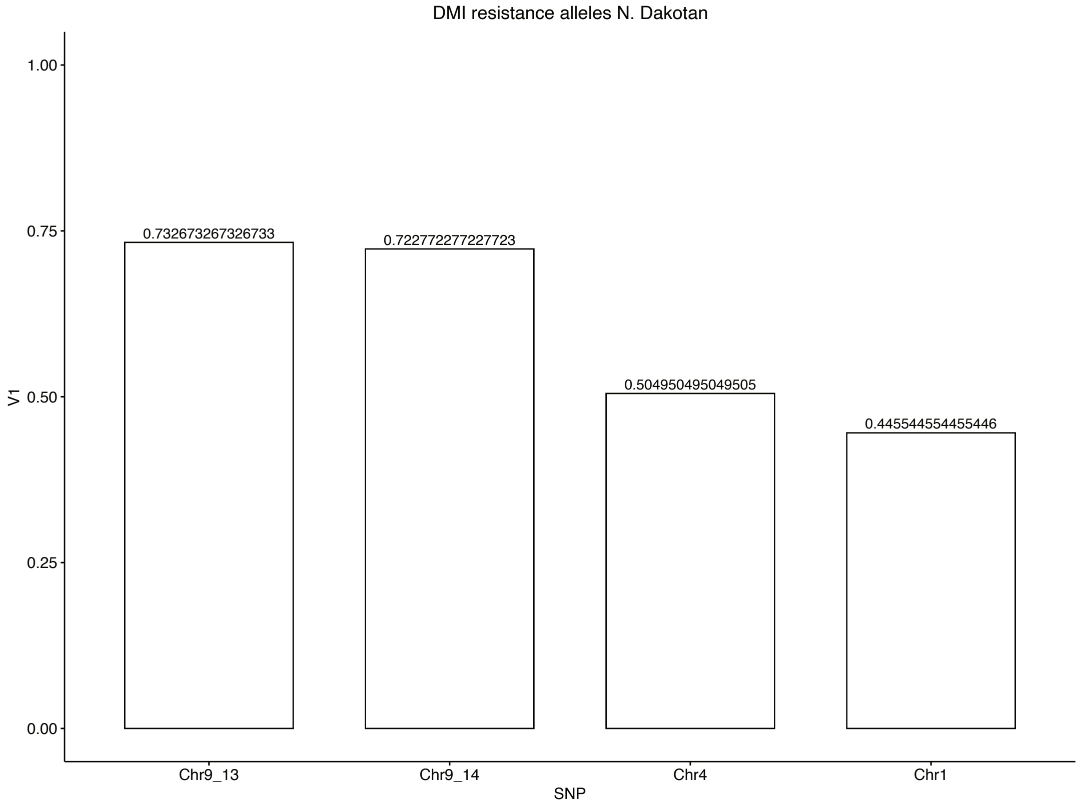

Figure S9: Frequency of DMI resistance associated markers in the N. Dakotan populaton

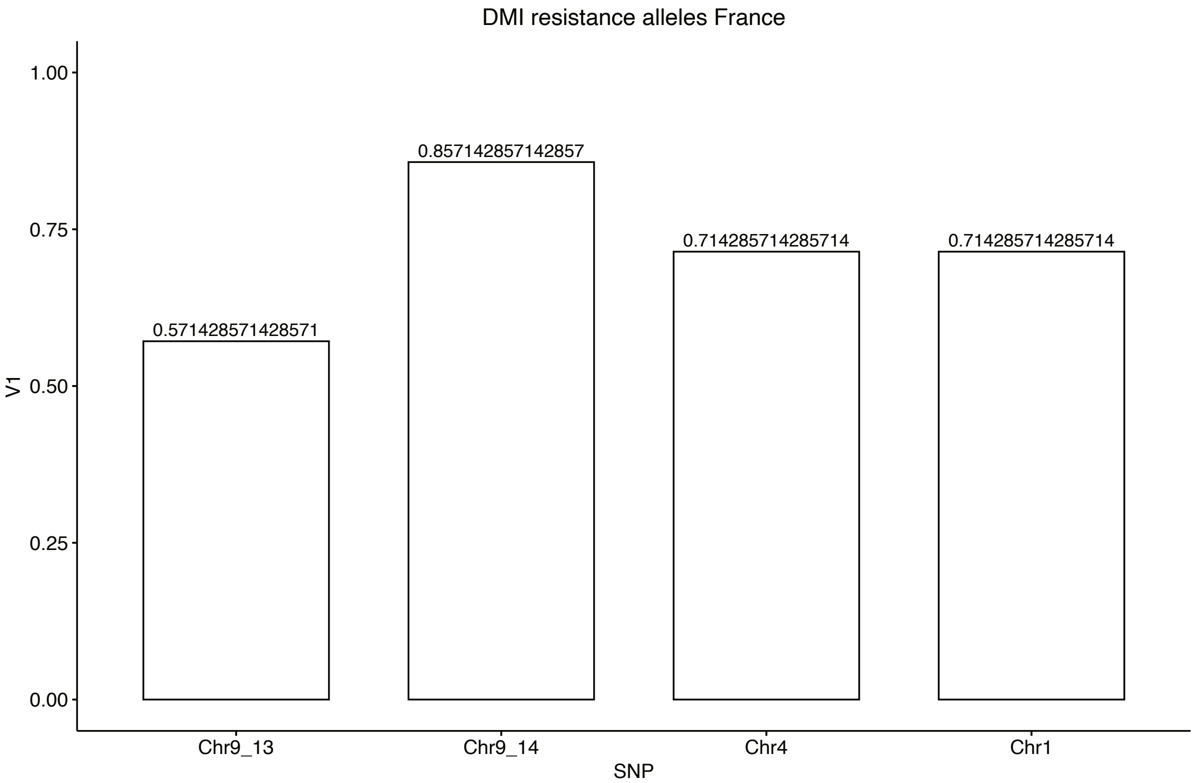

Figure S10: Frequency of DMI resistance associated markers in the French populaton

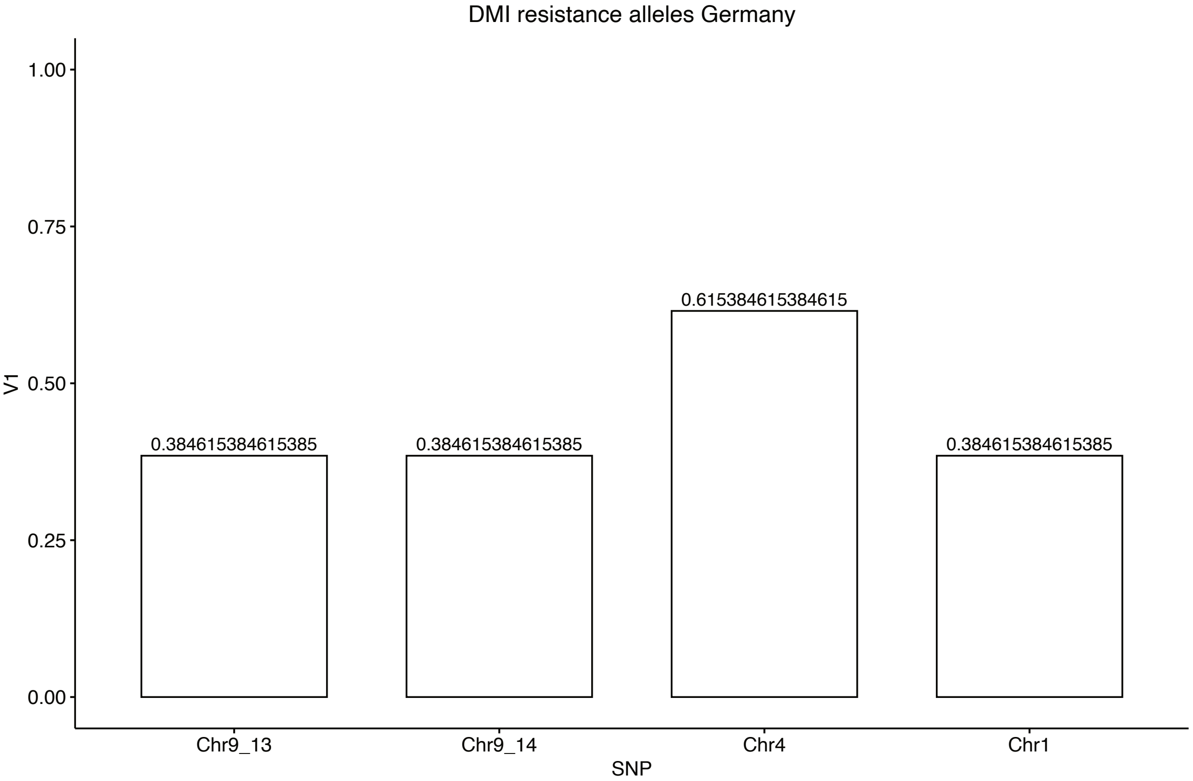

Figure S11: Frequency of DMI resistance associated markers in the German populaton

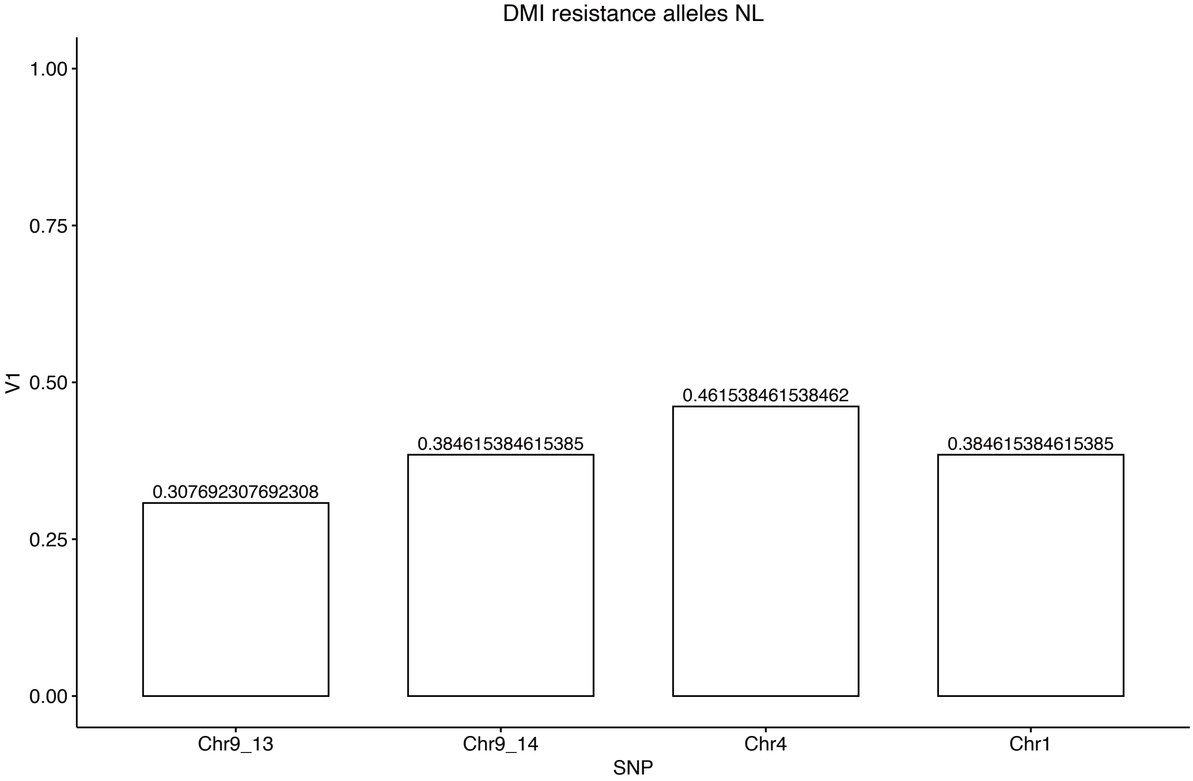

Figure S12: Frequency of DMI resistance associated markers in the Dutch populaton

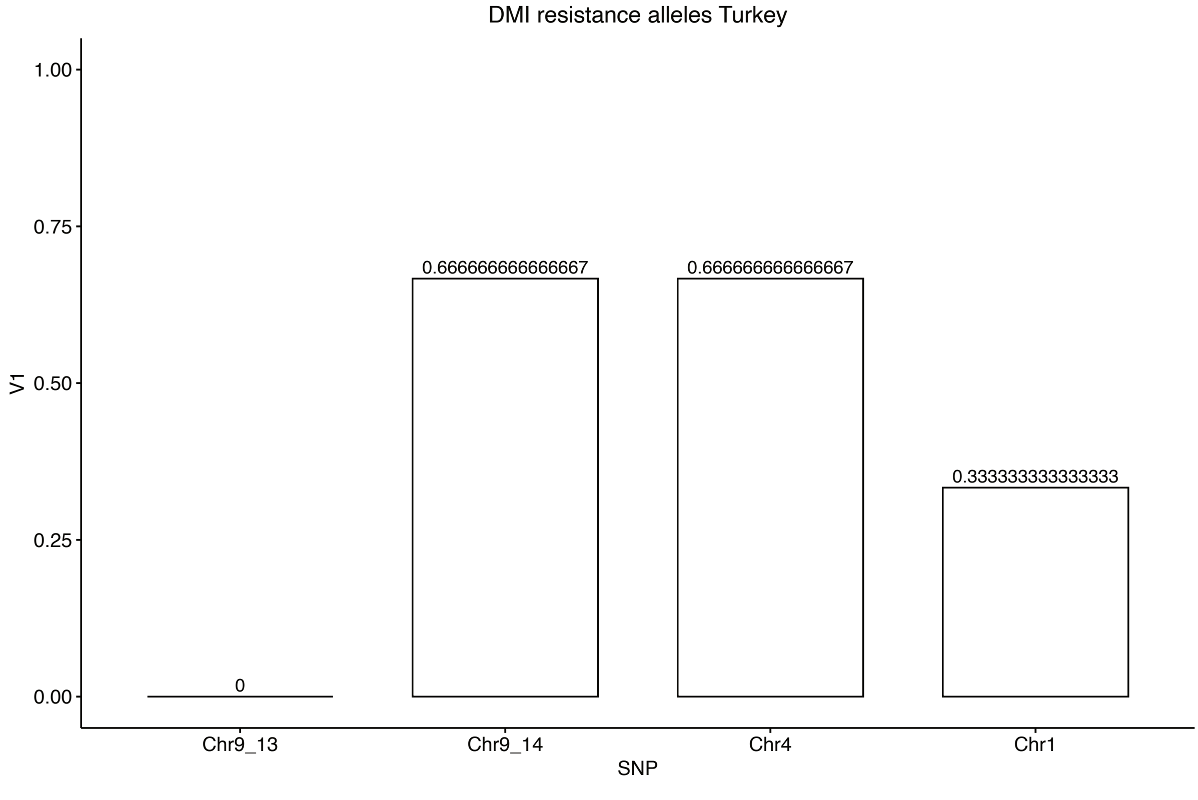

Figure S13: Frequency of DMI resistance associated markers in the Turkish populaton

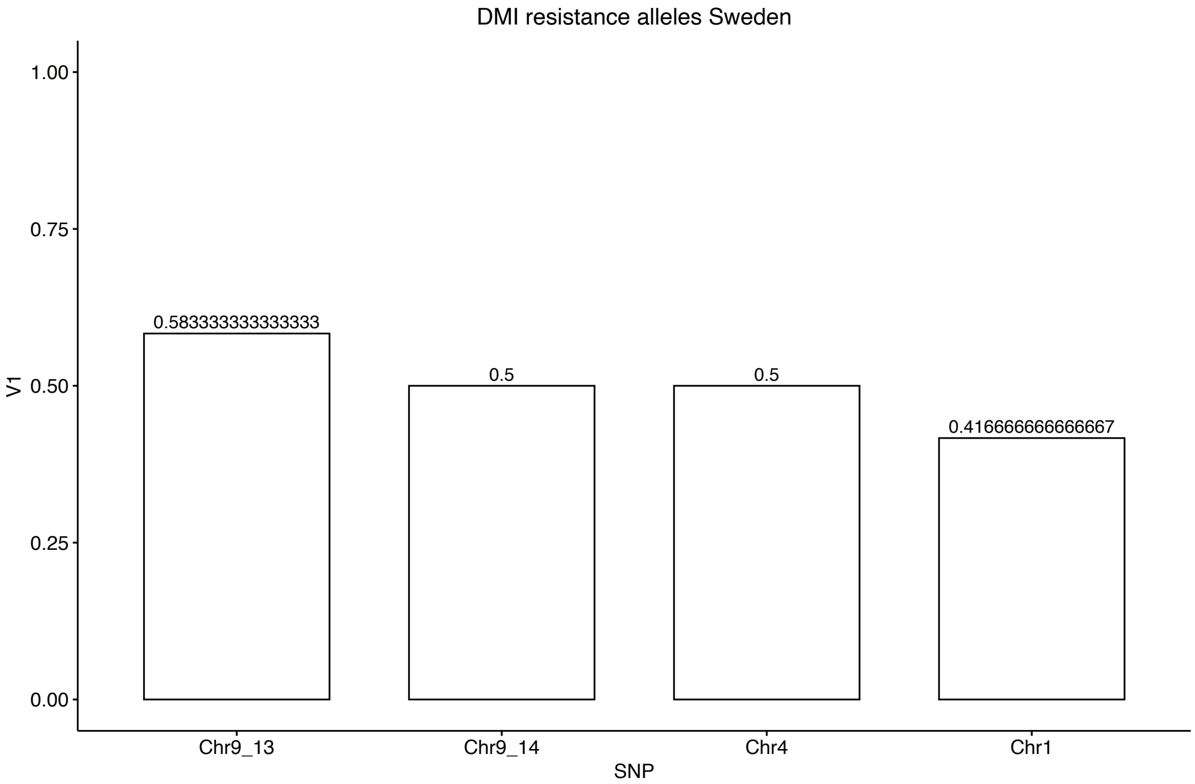

Figure S14: Frequency of DMI resistance associated markers in the Swedish populaton
